# Cytomegalovirus-induced T cell responses accelerate Alzheimer’s disease progression in mice

**DOI:** 10.1093/brain/awag043

**Published:** 2026-07-13

**Authors:** Morgan Marsden, James E McLaren, Ryan J Bevan, Daisy Penn-Ripley, Michelle Somerville, Sarah N Lauder, Manon H Jones, Lila-Blythe Maros, Matthew R McGurk, Awen Gallimore, David A Price, Kelly L Miners, Kristin Ladell, Florian A Siebzehnrubl, Timothy R Hughes, Ian R Humphreys, Mathew Clement

**Affiliations:** Division of Infection and Immunity, School of Medicine, Cardiff University, Cardiff CF14 4XN, UK; Systems Immunity Research Institute, School of Medicine, Cardiff University, Cardiff CF14 4XW, UK; Division of Infection and Immunity, School of Medicine, Cardiff University, Cardiff CF14 4XN, UK; Systems Immunity Research Institute, School of Medicine, Cardiff University, Cardiff CF14 4XW, UK; Dementia Research Institute, Cardiff University, Cardiff CF24 4HQ, UK; Division of Infection and Immunity, School of Medicine, Cardiff University, Cardiff CF14 4XN, UK; Systems Immunity Research Institute, School of Medicine, Cardiff University, Cardiff CF14 4XW, UK; Division of Infection and Immunity, School of Medicine, Cardiff University, Cardiff CF14 4XN, UK; Systems Immunity Research Institute, School of Medicine, Cardiff University, Cardiff CF14 4XW, UK; Division of Infection and Immunity, School of Medicine, Cardiff University, Cardiff CF14 4XN, UK; Systems Immunity Research Institute, School of Medicine, Cardiff University, Cardiff CF14 4XW, UK; Division of Infection and Immunity, School of Medicine, Cardiff University, Cardiff CF14 4XN, UK; Systems Immunity Research Institute, School of Medicine, Cardiff University, Cardiff CF14 4XW, UK; Division of Infection and Immunity, School of Medicine, Cardiff University, Cardiff CF14 4XN, UK; Systems Immunity Research Institute, School of Medicine, Cardiff University, Cardiff CF14 4XW, UK; Division of Infection and Immunity, School of Medicine, Cardiff University, Cardiff CF14 4XN, UK; Systems Immunity Research Institute, School of Medicine, Cardiff University, Cardiff CF14 4XW, UK; Division of Infection and Immunity, School of Medicine, Cardiff University, Cardiff CF14 4XN, UK; Systems Immunity Research Institute, School of Medicine, Cardiff University, Cardiff CF14 4XW, UK; Division of Infection and Immunity, School of Medicine, Cardiff University, Cardiff CF14 4XN, UK; Systems Immunity Research Institute, School of Medicine, Cardiff University, Cardiff CF14 4XW, UK; Division of Infection and Immunity, School of Medicine, Cardiff University, Cardiff CF14 4XN, UK; Systems Immunity Research Institute, School of Medicine, Cardiff University, Cardiff CF14 4XW, UK; Division of Infection and Immunity, School of Medicine, Cardiff University, Cardiff CF14 4XN, UK; Systems Immunity Research Institute, School of Medicine, Cardiff University, Cardiff CF14 4XW, UK; European Cancer Stem Cell Research Institute, School of Biosciences, Cardiff University, Cardiff CF24 4HQ, UK; Division of Infection and Immunity, School of Medicine, Cardiff University, Cardiff CF14 4XN, UK; Systems Immunity Research Institute, School of Medicine, Cardiff University, Cardiff CF14 4XW, UK; Division of Infection and Immunity, School of Medicine, Cardiff University, Cardiff CF14 4XN, UK; Systems Immunity Research Institute, School of Medicine, Cardiff University, Cardiff CF14 4XW, UK; Division of Infection and Immunity, School of Medicine, Cardiff University, Cardiff CF14 4XN, UK; Systems Immunity Research Institute, School of Medicine, Cardiff University, Cardiff CF14 4XW, UK

**Keywords:** virus, T cells, Alzheimer’s disease, herpesvirus, neurodegeneration, cognition

## Abstract

Infections have long been implicated as causative factors in Alzheimer’s disease (AD). Multiple studies have further suggested a key role for herpesviruses, such as cytomegalovirus (CMV).

Using transgenic 3xTg-AD mice, we demonstrate that systemic infection with the β-herpesvirus murine CMV (MCMV) accelerates the development of cognitive decline, tauopathy and synaptic loss in the hippocampus, all of which are key features of AD.

Accelerated disease progression after infection was associated with substantial lymphocyte infiltration into the brain, dominated by MCMV-specific effector memory CD8^+^ T cells expressing CXCR3. T cell receptor analyses revealed that clonally diverse virus-specific CD8^+^ T cells were selectively recruited into the brain during the development of AD. T cell depletion or treatment with the antiviral drug valganciclovir during chronic infection reduced lymphocytic infiltrates in the brain and reversed cognitive decline.

These data provide a mechanistic link between chronic viral infections and the development of AD.

## Teaser

Marsden *et al.* show that chronic cytomegalovirus infection accelerates cognitive decline, tauopathy and synaptic loss in an Alzheimer’s disease mouse model. Disease progression is associated with infiltration of virus-specific CD8+ T cells into the brain and can be reversed by antiviral treatment or T-cell depletion.

## Introduction

Alzheimer’s disease (AD) is the most common form of dementia, with ∼50 million cases worldwide and ∼152 million cases predicted by 2050.^1^ Although a number of genetic risk factors have been identified for early-onset and late-onset disease,^2^ it has remained unclear to what extent environmental factors impact the development and/or progression of AD.

The hypothesis that infectious diseases contribute to the development of AD was presented over a century ago by Alzheimer and Fischer, who noted pathological similarities with syphilitic dementia, caused by the obligate parasite *Treponema pallidum.*^3,4^ Since then, many bacteria, including *Borrelia spp.*,^5^ and viruses, including influenza^6^ and SARS-CoV-2,^7^ have also been implicated in the development of AD. Chronic human herpesviruses (HHVs) with neurotropic properties have received particular attention as potential risk factors for AD, and DNA from herpes simplex virus (HSV),^8,9^ HHV6-A and HHV-7^10^ has been found in the brains of patients suffering from AD. Moreover, the recombinant shingles vaccine, which targets varicella-zoster virus (VZV), has been associated with a lower dementia risk.^11^ Although not classically considered as neurotropic in the absence of immune compromise, the β-herpesvirus human cytomegalovirus (HCMV) has also been associated with accelerated cognitive decline and the development of AD.^12-15^ However, the mechanisms underlying these associations remain largely unknown, despite the potential of such information to reveal new treatment strategies for patients with AD.

Immune pathologies could feasibly link microbial infections with the development of AD.^16^ In line with this notion, T cells have been observed in the hippocampus, leptomeninges and CSF of patients with AD.^17-19^ Similar findings have been reported in mice.^19,20^ AD neuropathology is characterized by neuritic plaques and neurofibrillary tangles (NFTs), which develop as a consequence of amyloid-beta (Aβ) accumulation and tau phosphorylation, respectively, leading to synaptic loss and neuronal cell dysfunction. T cells associate with tau pathology in humans^17^ and drive tauopathy-induced neurodegeneration in mice.^21^ In addition, post-mortem studies have revealed the presence of T cells in the brains of patients with AD,^16^ and severe disease has been associated with CD8^+^ T cell infiltrates in the hippocampus,^17,19,22^ raising the possibility that host immune responses to intracellular infections could contribute to disease progression. In accordance with this hypothesis, studies of T cell infiltrates in the CSF of patients with AD have revealed the presence of clonally expanded CD8^+^ T cells reactive against the γ-herpesvirus Epstein-Barr virus (EBV).^18^

HCMV has also been implicated as a key contributor to the onset of AD.^12,14,23,24^ The associated mechanisms nonetheless remain obscure. In this study, we used the well-characterized murine cytomegalovirus (MCMV) experimental model of herpesvirus infection to investigate whether antiviral immune responses impact disease progression in 3xTg-AD mice, which harbour three genetic mutations associated with familial AD. Our data provide new evidence to support the proposition that herpesvirus-induced T cells drive infection-associated brain pathology and cognitive decline in AD.

## Materials and methods

### Ethics statement

All mouse experiments were performed at Cardiff University under UK Home Office Project Licence P8159A562.

### Mice and treatments

C57BL/6J WT mice were purchased from Envigo or Charles River (JAX strain #000664). B6129SF2/J mice were purchased from The Jackson Laboratory (JAX strain #101045). 3xTg-AD mice (B6;129-Tg(APPSwe,tauP301L)1Lfa *Psen1^tm1Mpm^*/Mmjax), which contain three genetic mutations associated with familial AD (APP Swedish, MAPT P301L and PSEN1 M146V), were purchased from The Jackson Laboratory (JAX strain #004807).^25^ Mice were infected with salivary gland-derived MCMV (Smith Strain) or mock-infected with phosphate-buffered saline (PBS) intraperitoneally (i.p.) as described in each figure. In some experiments, mice were treated with 1 mg/ml valganciclovir hydrochloride (Cardiff and Vale NHS Pharmacy) dissolved in animal drinking water (replaced weekly) from Day 60 post-infection. In other experiments, mice were treated with 100 μg each of depleting antibodies targeting CD4 (clones GK1.5 and YTS191, BioXCell) and CD8 (clones YTS156.7.7 and YTS169.4, BioXCell) or 400 μg of an isotype control antibody targeting keyhole limpet haemocyanin (InVivoMAb rat IgG2b, BioXCell) i.p. on Day 60 post-infection, repeated after 1 week (400 μg total per mouse).

### T cell receptor sequencing

Eight-to-ten-week-old female C57BL/6 and 3xTg-AD mice (*n* = 12 per group) were infected with 5 × 10^4^ pfu of MCMV (i.p). At 9 months of age, blood was extracted via cardiac puncture, and brains were harvested after whole-mouse perfusion with PBS. Leucocytes were isolated from blood and brain as described later and pooled into two groups for each phenotype and each tissue (*n* = 6 per group). Cells were stained with Zombie Aqua (BioLegend) and then with anti-CD16/CD32 Fc-block (BioLegend). MCMV-specific cells were identified using a phycoerythrin (PE)-conjugated H-2K^b^ tetramer (25 μg/ml) representing the SSPPMFRV epitope from M38 (residues 316–323, National Institutes of Health Tetramer Core Facility). Cells were stained with the tetramer for 15 min at 37°C and then with anti-CD3–BV605 (clone 145-2C11, BioLegend) and anti-CD8–APC (clone 53.6.7, BioLegend) for 20 min at 4°C. Data were acquired using a modified FACS Aria II (BD Biosciences). M38 tetramer^+^ CD8^+^ T cell populations were sorted into RNAprotect Buffer (Qiagen). T cell receptor (TCR) deep-sequencing was performed across all β-chain transcripts using a multiplex approach (iRepertoire). RNA was extracted and amplified using Amp2seq (arm-PCR). Samples were sequenced at a depth of approximately 125 000 reads per library using a Miseq Nano (Illumina).

### Animal behaviour

Animal behaviour and cognitive performance were measured using novel object recognition (NOR) as described previously.^26^ In each behavioural test, at least 4–10 mice were included per condition. Behavioural testing was performed using a custom-made plastic test arena (39 cm × 39 cm × 39 cm) with three non-transparent walls and one transparent wall for observation. The arena was placed in a class II laminar flow hood affixed with a HERO Session Camera (GoPro). Each animal was tested in isolation. Briefly, each mouse was placed in the box with two identical objects (familiar objects, FOs) for 10 min, returned to its home cage for 20 min, and then placed in the box again for another 10 min with one FO and one novel object (NO). The FOs and NOs were similar in size but differed in colour and shape. All recorded data were analysed using Ethovision XT 13 Video Tracking Software (Noldus). Discrimination ratios (DRs) were determined by dividing the time spent exploring the NO (physical touching of object) by the time spent exploring both the FO and the NO. Impairment of cognition was assigned at a DR below 0.5. The analyst was blinded to group allocation. Spontaneous alternation rate (AR) behaviour was assessed via a T-maze test using a custom-made plastic test arena with enclosed opaque sides as described previously.^27^ Mice were not habituated to the test arena before entering the maze. All partition doors were raised initially. The animal was placed into position A and allowed to choose a goal arm. Once a side was chosen, the partition door was closed to confine the animal for 30 s, after which the animal was removed from the confined area (ensuring little stress), and the partition was raised again. The animal was then returned to position A. This procedure was repeated to capture a total of 10 binary choices per mouse. Each test took a minimum of 1 min. The AR was recorded as the number of times each mouse chose the same goal arm (%).

### Viral load quantification

Organ-specific infectious virus was quantified via plaque assay as described previously.^28^ Viral DNA copy number in saliva was determined via qPCR for relative expression of IE1 using the forward primer 5′-AGCCACCAACATTGACCACGCAC-3′ and the reverse primer 5′-GCCCCAACCAGGACACACAACTC-3′.^29,30^ Genomic DNA was isolated from brain, lung and spleen tissue using a DNeasy Blood and Tissue Kit (Qiagen). MCMV IE1 was assayed via qPCR using a QuantStudio 12K Flex Real-Time PCR System (Applied Biosystems) with iTaq Universal SYBR Green Platinum Supermix (Bio-Rad). Each reaction was based on 100 ng of DNA. The forward primer sequence was 5′-TCAGCCATCAACTCTGCTACCAAC-3′, and the reverse primer sequence was 5′-ATCTGAAACAGCCGTATATCATCTTG-3′. Viral genome copies were calculated using a standard curve constructed from the plasmid pARK25 MCMV (limit of detection = 10 copies).

### Cell phenotyping

Blood, brain and spleen leucocytes from female C57BL/6 and 3xTg-AD mice were stained as described previously.^29,31^ Blood was extracted via cardiac puncture, and mice were subsequently perfused with PBS. Harvested brains were cut into small pieces using dissection scissors and incubated in RPMI 1640 medium (Thermo Fisher Scientific) supplemented with 5 mM CaCl_2_ (Sigma-Aldrich), 5% fetal calf serum (Thermo Fisher Scientific), 1 mg/ml collagenase D (Roche Diagnostics) and 10 mg/ml DNAse I (Sigma-Aldrich) for 45 min at 37°C. Leucocytes were isolated using a cell strainer (40 μm) and purified over Percoll (GE Healthcare). Cells were stained with Zombie Aqua (BioLegend) and then with anti-CD16/CD32 Fc-block (BioLegend). Tetramer staining was performed as described earlier for the H-2K^b^ SSPPMFRV epitope from M38 (residues 316–323), the H-2K^b^ RALEYKNL epitope from IE3 (residues 416-423), the H-2D^b^ HGIRNASFI epitope from M45 (residues 985–993) and the H-2K^b^ TVYGFCLL epitope from m139 (residues 419–426) (National Institutes of Health Tetramer Core Facility). Cells were then stained with combinations of the following directly conjugated monoclonal antibodies: anti-CCR9–FITC (clone 9B1, BioLegend), anti-CD3–PE-Cy7 (clone 53.6.7, BioLegend), anti-CD4–BV605, anti-CD4–PE-Cy7 or anti-CD4–PerCP-Cy5.5 (clone RM4-5, BioLegend), anti-CD8–APC-Cy7, anti-CD8–BV605, anti-CD8–BV711, anti-CD8–PE-Cy7 or anti-CD8–PerCP-Cy5.5 (clone 53.6.7, BioLegend), anti-CD11a–PerCP-Cy5.5 (clone M17/4, BioLegend), anti-CD27–PE (clone LG749, eBiosciences), anti-CD44–APC-Cy7, anti-CD44–FITC or anti-CD44–PerCP (clone IM7, BioLegend), anti-CD45–BV605 (clone 30-F11, BioLegend), anti-CD62L–BV711, anti-CD62L–FITC or anti-CD62L–PE-Cy7 (clone MEL-14, BioLegend), anti-CD69–BV711 or anti-CD69–PE-Cy7 (clone H1.2F3, BioLegend), anti-CD103–PE-Dazzle (clone 2E7, BioLegend), anti-CD127–BV711 (clone A7R34, BioLegend), anti-CX3CR1–APC-Cy7 (clone SA011F11, BioLegend), anti-CXCR3–AF488 (clone CXCR3-173, BioLegend), anti-Ly49H–FITC (clone 3D10, BioLegend), anti-NK1.1–PE (clone PK136, BioLegend) and anti-NKp46–BV605 (clone 29A1.4, BioLegend) for 20 min at 4°C. Data were acquired using an Attune NxT flow cytometer (Thermo Fisher Scientific) and analysed using FlowJo version 10 (FlowJo LLC).

### DiOlistic spine labelling

Freshly dissected and sectioned hippocampal slices (200 μm, McIlwain Tissue Chopper) were analysed using DiOlistic labelling to visualize dendritic and synaptic architecture.^32^ Briefly, tungsten particles (1.67 µm, Bio-Rad) were coated with 1,1′-dioctadecyl-3,3,3′,3′-tetramethylindocarbocyanine perchlorate (DiI, Thermo Fisher Scientific) dissolved in dichloromethane (Sigma-Aldrich). DiI-coated particles were loaded in ethylene tetrafluoroethylene tubing and delivered onto the tissue slices through a cell culture insert (3 μm) at 100–120 psi using a Helios Gene Gun (Bio-Rad). Dye diffusion was facilitated in Neurobasal-A Medium (Thermo Fisher Scientific) for 20 min at 37°C in a 5% CO_2_ atmosphere. Nuclei were stained with Hoechst 33342 (Thermo Fisher Scientific). Tissue slices were then fixed with 4% paraformaldehyde for 30 min at room temperature and mounted in FluorSave Reagent (Sigma-Aldrich). Dendritic spines on CA1 hippocampal apical dendrites were imaged using an SP8 LIGHTNING Confocal Microscope (Leica Microsystems). Segments longer than 30 μm were reconstructed in 3D using Imaris Filament Tracer version 9.3.1 (Bitplane), and spines were subtyped morphologically using Spine Classifier (MATLAB).^32^ The following predetermined settings were used for classification: <0.8 μm, ‘stubby’ spines; 0.8–3 μm with a head diameter greater than neck width, ‘mushroom spines’; 0.8–3 μm, ‘thin’ spines. Each measurement was taken from at least four separate values for each mouse, and each reconstructed dendrite segment was verified manually.

### Immunohistochemistry

Brains were excized and fixed with 10% neutral-buffered formalin saline after whole-mouse perfusion with PBS. Whole brains were embedded in paraffin and sectioned at a thickness of 5 µm. Sections were stained using an automated system with BOND RX (Leica Biosystems). Antigen retrieval was performed using BOND ER2 Solution (Leica Biosystems). Endogenous peroxidase activity was quenched using 1% H_2_O_2_ (Sigma-Aldrich). Non-specific binding was blocked using 10% goat serum (Sigma-Aldrich). Sections were then incubated with anti-CD3 (clone ARC51750, ABclonal) and anti-CD8 (clone D4W2Z, Cell Signaling Technology) for 50 min at room temperature. Bound primary antibodies were detected using Rabbit IgG VisUCyte (Bio-Techne) or ImmPRESS HRP Goat Anti-Rat IgG Polymer (Vector Labs), followed by incubation with Tyramide Conjugates (Biotium) to generate fluorescence signals at 405, 488 and 594 nm. Nuclei were stained with Hoechst 33342 (Thermo Fisher Scientific). Slides were mounted using Vectashield Vibrance (Vector Labs) and imaged at ×20 magnification using an Axio Scan.Z1 Slide Scanner (Zeiss) with a Plan-Apochromat Objective 20×/0.8 NA (Zeiss). The number of CD3^+^/CD8^+^ cells was quantified using QuPath version 0.5.1 (University of Edinburgh).^33^

### ELISAs

Perfused brains were homogenized mechanically. Supernatants were analysed using a Mouse IL-18 ELISA (BioLegend), a Human Total Tau ELISA (BioLegend), a Human Tau (phospho-T231) ELISA (Thermo Fisher Scientific) and a Human Aβ_42_ ELISA (Abcam). Brain homogenates were further homogenized using Tris/EDTA buffer pH 9 (Qiagen), 1% Triton X-100 (Sigma-Aldrich), 0.1% NaN_3_ and 1× Protease Inhibitor Cocktail (Sigma-Aldrich) and analysed using a Human Aβ_40_ ELISA (Thermo Fisher Scientific).

### Statistics

Significance was assessed across two groups using the Mann–Whitney U-test, Welch’s *t*-test or the Wilcoxon matched pairs test and across more than two groups using a one-way or two-way ANOVA with Tukey’s *post hoc* correction. Survival curves were analysed using the Mantel–Cox test. Independent biological replicates were performed as detailed for each figure. Outliers are included and shown in all datasets.

## Results

### Chronic MCMV infection accelerates cognitive decline in 3xTg-AD mice

To investigate how herpesviruses could impact the development of AD, either directly or indirectly via the induction of antiviral immunity, we first established an experimental model using adult 3xTg-AD mice infected with MCMV. B6129SF2/J and C57BL/6 mice have been used previously to control for the mixed genetic background of 3xTg-AD mice.^34,35^ However, C57BL/6 mice are relatively resistant to MCMV infection, reflecting expression of the activating natural killer (NK) cell receptor Ly49H.^36,37^ 3xTg-AD mice exhibited comparable survival (Supplementary Fig. 1A), weight loss (Supplementary Fig. 1B), control of acute (Supplementary Fig. 1C) and chronic viral replication (Supplementary Fig. 1D), and virus shedding (Supplementary Fig. 1E) to C57BL/6 mice, whereas B6129SF2/J mice were more susceptible to the pathogenic effects of MCMV (Supplementary Fig. 1A–D). Accordingly, NK cells from B6129SF2/J mice expressed Ly49H less frequently than NK cells from C57BL/6 or 3xTg-AD mice (Supplementary Fig. 2A) and, consistently, mounted weaker NK cell responses during acute MCMV infection (Supplementary Fig. 2B). C57BL/6 mice were therefore chosen as the appropriate comparator strain for 3xTg-AD mice.

**Figure 1:**
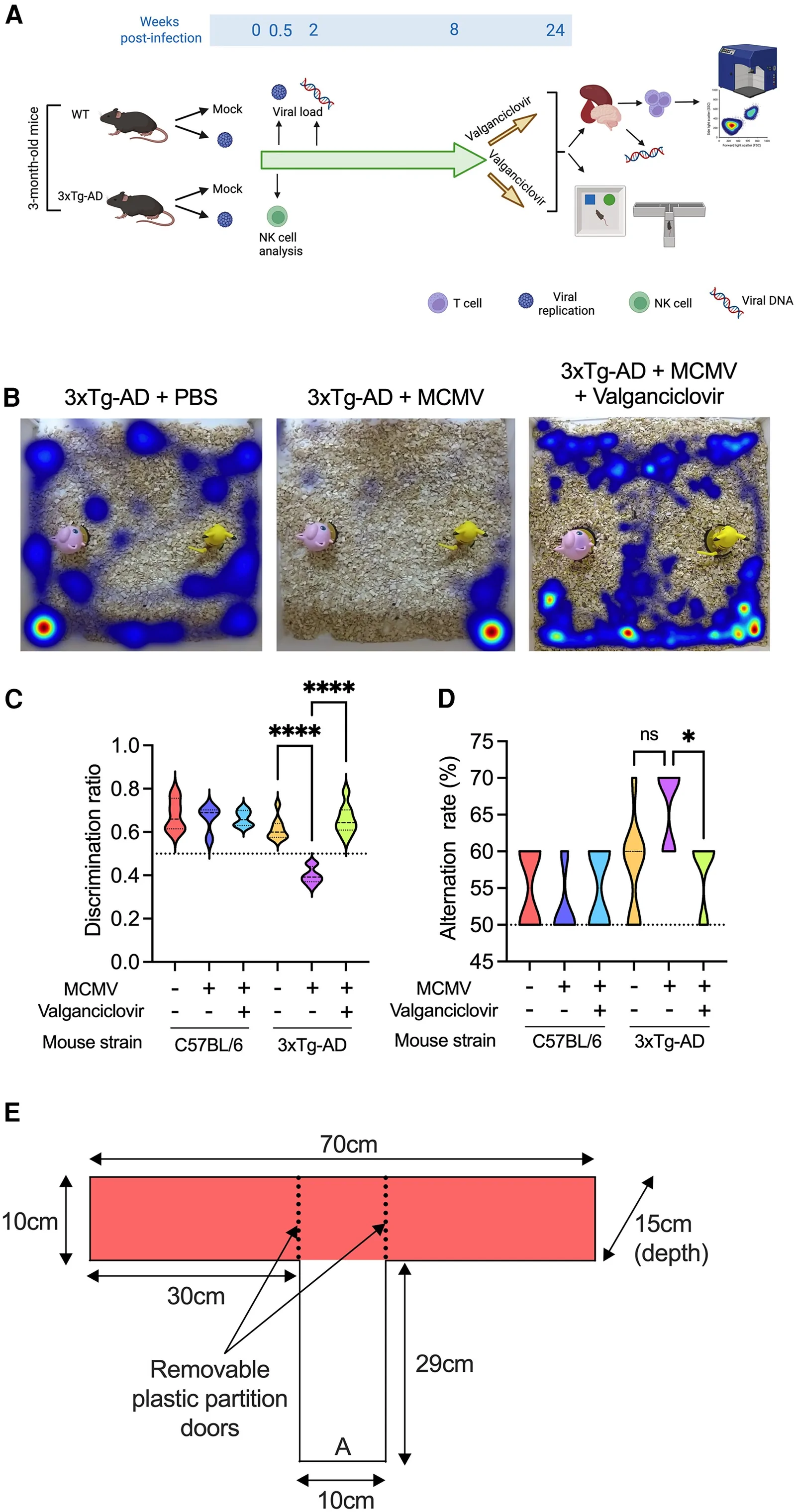
**MCMV infection exacerbates cognitive decline in 3xTg-AD mice.** (**A**) Schematic representation of the study design. Created in BioRender. Humphreys, I. (2026) https://BioRender.com/tupgv6j. (**B**–**D**) C57BL/6 and 3xTg-AD mice were mock-infected with PBS or infected with 5 × 10^4^ pfu of MCMV (i.p.). Some groups of mice were treated with 1 mg/ml valganciclovir hydrochloride dissolved in animal drinking water continuously from Day 60 post-infection. Animal behaviour and cognitive performance were assessed 6 months post-infection using novel object recognition (NOR) (**B** and **C**) and spontaneous alternation rate (AR) (**D**). (**B**) Heat map analysis tracking animal path and animal behaviour for 3xTg-AD mice. (**C**) Post-NOR assessment of discrimination ratio (DR). Impaired cognition is represented by a DR below 0.5. (**D**) Post-T-maze test assessment of AR replicated 10 times/mouse/condition. Impaired cognition is represented by an AR above 60% per chosen open arm. Data are representative of 2–5 independent experiments with *n* = 4 C57BL/6 and *n* = 10 3xTg-AD mice/group. Significance was assessed using a two-way ANOVA with Tukey’s *post hoc* correction. *P*-values are reported as follows: n.s. = *P* > 0.05, *P ≤ 0.05, **P ≤ 0.01, ***P ≤ 0.001, ****P ≤ 0.0001. (**E**) Schematic representation of the T-maze apparatus used for spontaneous alternation rate testing. AD = Alzheimer's disease; i.p. = intraperitoneal; MCMV = murine cytomegalovirus.

MCMV can persist in the brains of neonatal mice but is rarely detectable in the brains of immunocompetent adult mice.^38,39^ In line with this dichotomy, we detected comparable viral loads in the lungs and spleens of chronically infected C57BL/6 versus 3xTg-AD mice during latency (Supplementary Fig. 3A and B), whereas the corresponding brain tissues contained no detectable MCMV DNA (Supplementary Fig. 3C). To relate these observations to the development of AD, we infected 2–3-month-old female C57BL/6 or 3xTg-AD mice with MCMV. After 6 months, mock-infected 3xTg-AD mice exhibited comparable cognition to mock-infected C57BL/6 mice, both in terms of NO recognition (NOR) and T-maze AR (Fig. 1A–D). In contrast, MCMV-infected 3xTg-AD mice exhibited impaired NOR, indicated by reduced DRs, and altered spatial working memory, indicated by reduced ARs, compared with mock-infected or MCMV-infected C57BL/6 mice and mock-infected 3xTg-AD mice (Fig. 1A–D). Moreover, treatment with valganciclovir hydrochloride, which exerts robust antiviral activity against MCMV,^40^ during the chronic phase of infection (from Day 60) reversed all measures of virus-associated cognitive decline (Fig. 1A–D).

These results suggest that chronic MCMV infection accelerates cognitive decline in 3xTg-AD mice.

### MCMV infection induces neuronal damage and tauopathy in 3xTg-AD mice

**Figure 2:**
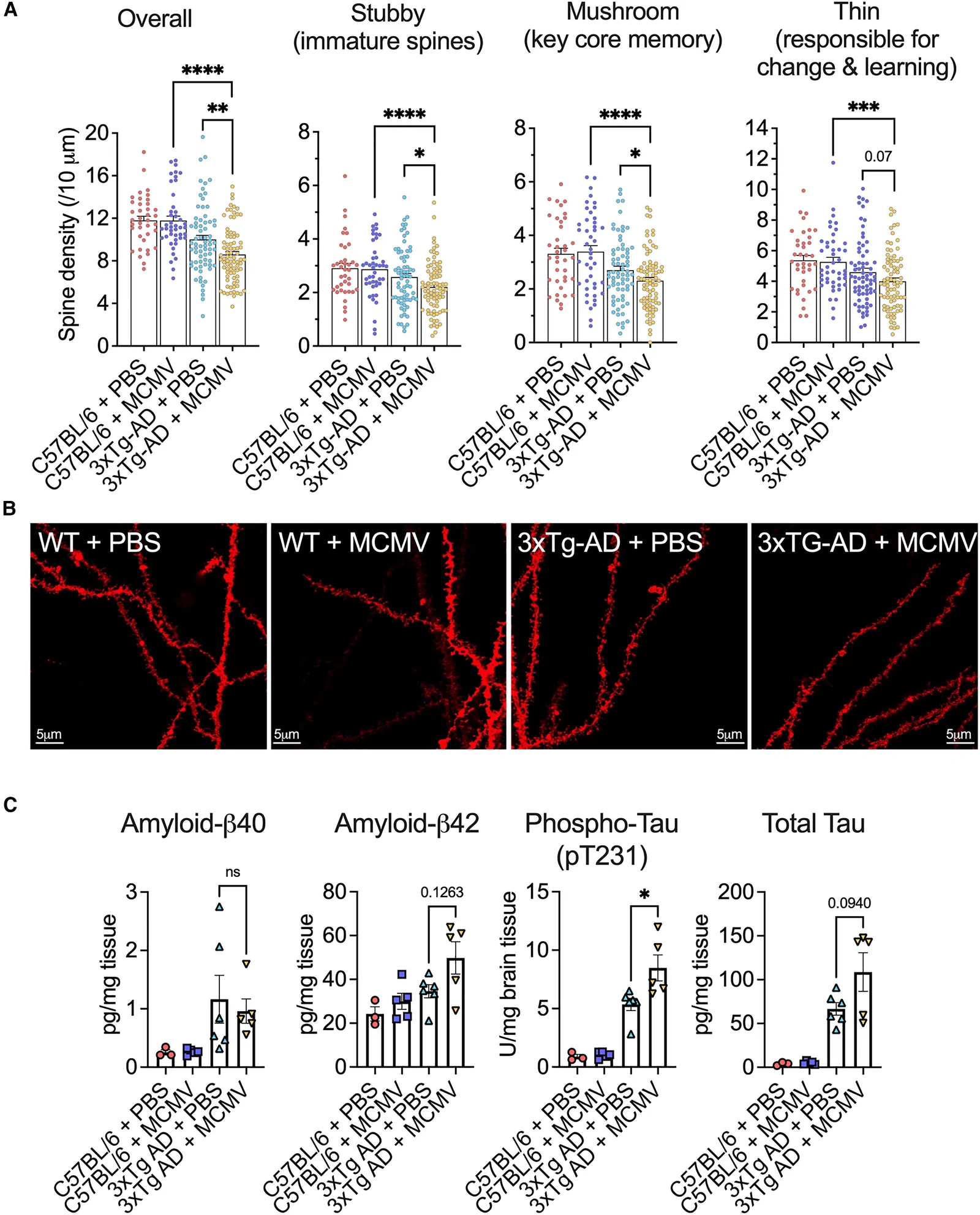
**MCMV infection promotes loss of neuronal function in 3xTg-AD mice.** C57BL/6 and 3xTg-AD mice were mock-infected with PBS or infected with 5 × 10^4^ pfu of MCMV (i.p.). Brains were harvested 6 months post-infection. (**A**) Overall and morphologically classified dendritic spine densities from the hippocampal CA1 region. Data are shown as mean ± standard error of the mean. Multiple data points are shown from *n* = 4 C57BL/6 and *n* = 6 3xTg-AD mice/group. Significance was assessed using Welch’s *t*-test. (**B**) Representative confocal images of dendritic spines from the hippocampal CA1 region. (**C**) Amyloid-β (Aβ)_40_, Aβ_42_, phospho-tau (pT231) and total tau concentrations in brain homogenates measured via ELISA. Data are representative of *n* = 3–5 C57BL/6 and *n* = 5–6 3xTg-AD mice/group. Significance was assessed using a one-way ANOVA with Tukey’s *post hoc* correction. *P*-values are reported as follows: n.s. = *P* > 0.05, *P = ≤0.05, **P ≤ 0.01, ***P ≤ 0.001, ****P ≤ 0.0001. AD = Alzheimer's disease; i.p. = intraperitoneal; MCMV = murine cytomegalovirus; WT = wild-type.

To understand the neuropathological mechanisms underpinning MCMV-induced cognitive decline in 3xTg-AD mice, we first investigated synaptic plasticity by analysing CA1 hippocampal dendritic spines 6 months after infection of 2–3-month-old female C57BL/6 or 3xTg-AD mice with MCMV. At this time, mock-infected 3xTg-AD mice exhibited lower spine densities compared with mock-infected C57BL/6 mice, albeit without achieving significance, and MCMV-infected 3xTg-AD mice exhibited substantially lower spine densities compared with mock-infected or MCMV-infected C57BL/6 mice and mock-infected 3xTg-AD mice (Fig. 2A and B). Morphological analyses further indicated a preferential loss of ‘stubby’ (common in early development and may represent a transient immature form) and ‘mushroom’ (long-term memory storage and synaptic connections) versus ‘thin’ spines (learning and plasticity) in 3xTg-AD mice infected with MCMV (Fig. 2A and B).^41,42^

AD neuropathology is characterized by neuritic plaques and NFTs, which develop as a consequence of Aβ accumulation and tau phosphorylation, respectively. Aβ plaques are generally only detectable from 12 months of age onwards in female 3xTg-AD mice.^43^ We nonetheless observed elevated concentrations of Aβ_42_ in brain homogenates from younger female 3xTg-AD mice 6 months after infection with MCMV (Fig. 2C). HCMV seropositivity associates with NFTs in individuals with AD.^23^ In addition, MCMV induces tau accumulation, at least *in vitro.*^44^ In line with these observations, we found elevated concentrations of phosphorylated and, to a lesser extent, total tau in brain homogenates from female 3xTg-AD mice 6 months after infection with MCMV, paralleling the changes in Aβ_42_ (Fig. 2C).

These data suggest that MCMV-induced cognitive decline is associated with hippocampal neuronal damage and the accumulation of Aβ_42_ and phosphorylated tau protein.

### MCMV induces brain-infiltrating CD4^+^ and CD8^+^ T cell responses in 3xTg-AD mice

**Figure 3:**
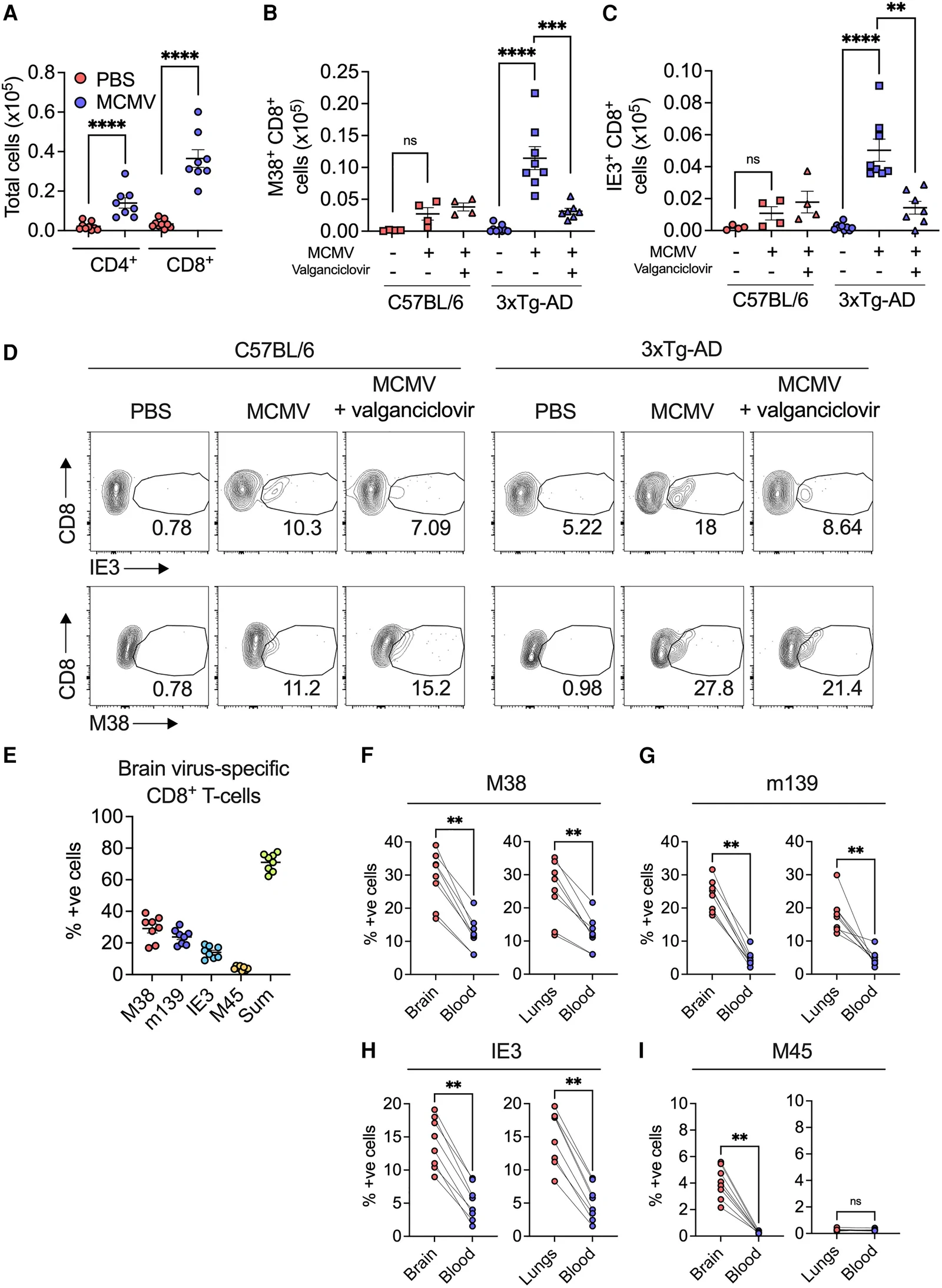
**MCMV infection promotes the accumulation of T cell infiltrates in the brains of 3xTg-AD mice.** C57BL/6 and 3xTg-AD mice were mock-infected with PBS or infected with 5 × 10^4^ pfu of MCMV (i.p.). Some groups of mice were treated with 1 mg/ml valganciclovir hydrochloride dissolved in animal drinking water continuously from Day 60 post-infection. Leucocytes were harvested from the indicated tissues 6 months (**A**–**D**) or 4 months post-infection (**E**–**I**). (**A**–**D**) Total CD4^+^ and CD8^+^ T cells (**A**) and MCMV tetramer^+^ CD8^+^ T cells (**B**–**D**) quantified via flow cytometry. Significance was assessed using the Mann–Whitney U-test (**A**) or a two-way ANOVA with Tukey’s *post hoc* correction (**B** and **C**). (**E**) MCMV tetramer^+^ CD8^+^ T cells in the brains of 3xTg-AD mice quantified via flow cytometry. (**F**–**I**) MCMV tetramer^+^ CD8^+^ T cells in the blood, brains and lungs of 3xTg-AD mice quantified via flow cytometry. Significance was assessed using the Wilcoxon matched pairs test. *P*-values are reported as follows: n.s. = *P* > 0.05, *P ≤ 0.05, **P ≤ 0.01, ***P ≤ 0.001, ****P ≤ 0.0001. AD = Alzheimer's disease; i.p. = intraperitoneal; MCMV = murine cytomegalovirus.

MCMV infection of C57BL/6 mice induces robust CD4^+^ and CD8^+^ T cell responses that often increase in magnitude over time.^45-47^ T cells have also been associated with tau pathology.^17,21^ Accordingly, we quantified CD4^+^ and CD8^+^ T cells in brain homogenates from MCMV-infected mice, where we were unable to detect viral DNA. We observed a marked accumulation of CD4^+^ and, more strikingly, CD8^+^ T cells in the brains of female 3xTg-AD mice 6 months after infection with MCMV (Fig. 3A). Moreover, high frequencies of these brain-infiltrating CD8^+^ T cells were specific for peptide epitopes derived from the IE3, M38 and m139 proteins and, to a lesser extent, the M45 protein encoded by MCMV (Fig. 3B–E). These virus-specific populations comprised ∼75% of all brain-infiltrating CD8^+^ T cells in MCMV-infected 3xTg-AD mice (Fig. 3E). The real figure may even approach totality, given that we did not measure all specificities for MCMV.^48^ In contrast, very few virus-specific CD8^+^ T cells were detected in brain homogenates from MCMV-infected C57BL/6 mice (Fig. 3B and C), consistent with the findings of an earlier study.^39^ Paired analyses further revealed that CD8^+^ T cells specific for IE3, M38 and m139 were enriched in brain and lung tissue versus blood from MCMV-infected 3xTg-AD mice, whereas CD8^+^ T cells specific for M45 were enriched in brain but not lung tissue versus blood from MCMV-infected 3xTg-AD mice (Fig. 3F–I).

These findings show that CD4^+^ and, to a greater extent, CD8^+^ T cells, many of which are specific for immunodominant viral epitopes derived from IE3, M38, m139 and M45, infiltrate the brains of MCMV-infected 3xTg-AD mice.

### Brain-infiltrating CD8^+^ T cells are highly differentiated and express CXCR3

**Figure 4:**
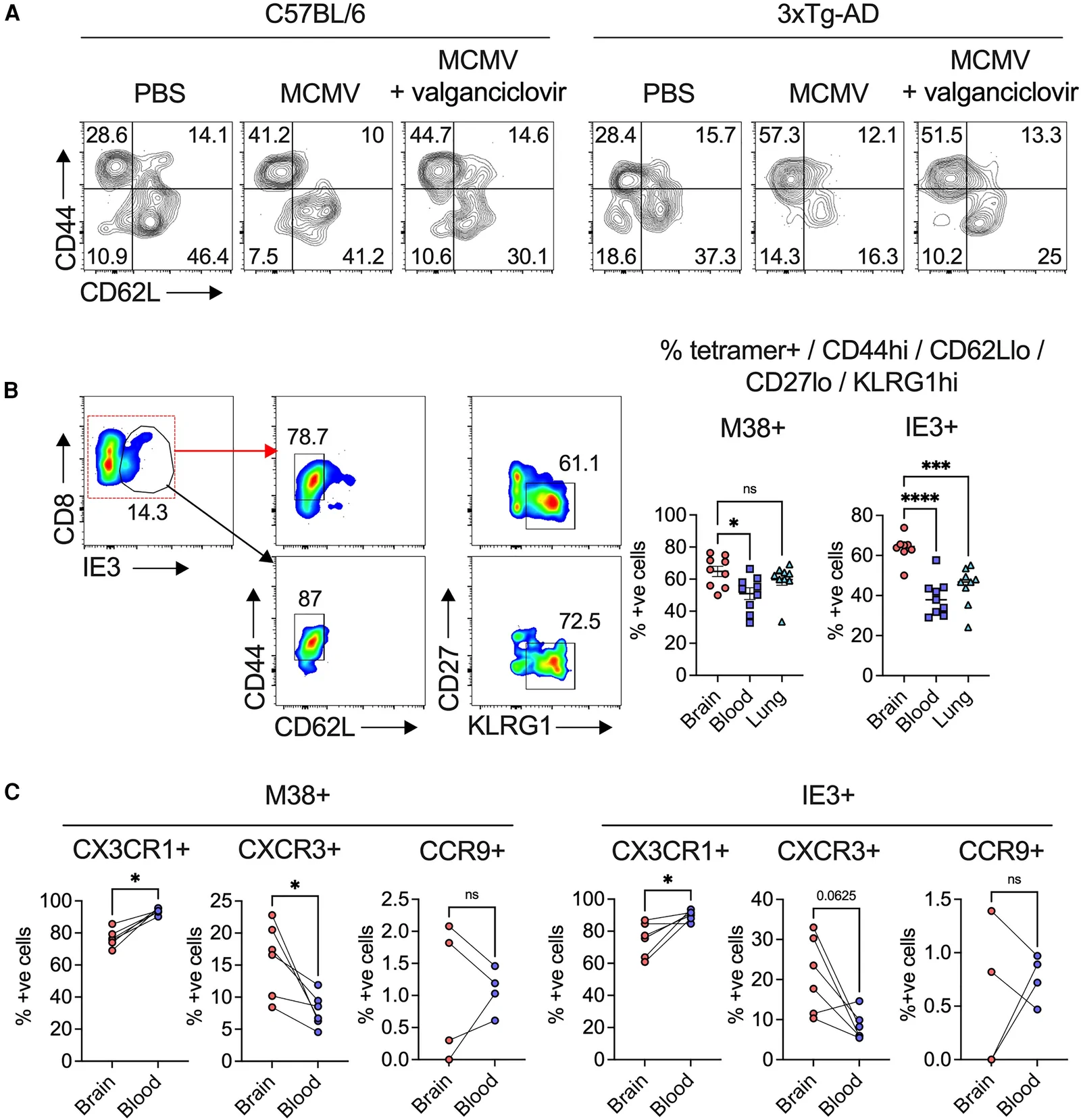
**MCMV infection drives the accumulation of brain-infiltrating effector memory CD8^+^ T cells in 3xTg-AD mice.** C57BL/6 and 3xTg-AD mice were mock-infected with PBS or infected with 5 × 10^4^ pfu of MCMV (i.p.). Leucocytes were harvested from the indicated tissues 6 months (**A**) or 4 months post-infection (**B** and **C**). (**A**) Bivariate flow cytometry plots showing percentage expression of CD44 versus CD62L among CD8^+^ T cells in the brains of C57BL/6 and 3xTg-AD mice. (**B**) Bivariate flow cytometry plots (*left*) and data summary (*right*) showing the phenotypic characteristics of IE3 tetramer^+^ CD8^+^ T cells in the blood, brains and lungs of 3xTg-AD mice. (**C**) Paired analyses of CX3CR1, CXCR3 and CCR9 expression among M38 tetramer^+^ (*left*) and IE3 tetramer^+^ CD8^+^ T cells (*right*) in the blood versus brains of 3xTg-AD mice. Data are representative of two independent experiments with *n* = 4 C57BL/6 and *n* = 9–10 3xTg-AD mice/group (**A**) or *n* = 4–6 3xTg-AD mice/group. Significance was assessed using a two-way ANOVA with Tukey’s *post hoc* correction (**B**) or the Wilcoxon matched pairs test (**C**). *P*-values are reported as follows: n.s. = *P* > 0.05, *P ≤ 0.05, **P ≤ 0.01, ***P ≤ 0.001, ****P ≤ 0.0001. AD = Alzheimer's disease; i.p. = intraperitoneal; MCMV = murine cytomegalovirus.

Immunophenotypic characterization revealed that MCMV infection was associated with an enrichment for brain-infiltrating CD8^+^ effector memory T (T_EM_) cells (CD44^+^/CD62L^−^), especially in 3xTg-AD mice (Fig. 4A). In contrast, very few naive CD4^+^ or CD8^+^ T cells (CD44^−^CD62L^+^CD127^+^) were present in the brains of mock-infected or MCMV-infected 3xTg-AD mice (Supplementary Fig. 4A), and MCMV infection did not induce increased frequencies of tissue-resident memory (T_RM_) CD4^+^ (CD11a^+^CD69^+^) or CD8^+^ T cells (CD69^+^CD103^+^) in the brains of C57BL/6 or 3xTg-AD mice (Supplementary Fig. 4B). Brain-infiltrating CD8^+^ T cells specific for IE3 or M38 exhibited a highly differentiated effector memory phenotype (CD44^hi^CD62L^lo^CD27^lo^KLRG-1^hi^) in 3xTg-AD mice, which was enriched relative to the corresponding virus-specific CD8^+^ T cells in lung tissue and blood from 3xTg-AD mice (Fig. 4B). MCMV-specific CD8^+^ T cells also expressed CXCR3 but not CCR9 or CX3CR1 more frequently in brain tissue versus blood from 3xTg-AD mice (Fig. 4C and Supplementary Fig. 4C). All of these chemokine receptors have been implicated in the pathogenesis of AD.^49^

These results show that MCMV drives highly differentiated CD8^+^ T cell responses in the brains of 3xTg-AD mice, potentially involving the CXCR3 ligand CXCL10.

### MCMV-specific CD8^+^ T cells in brain tissue are clonally diverse

**Figure 5:**
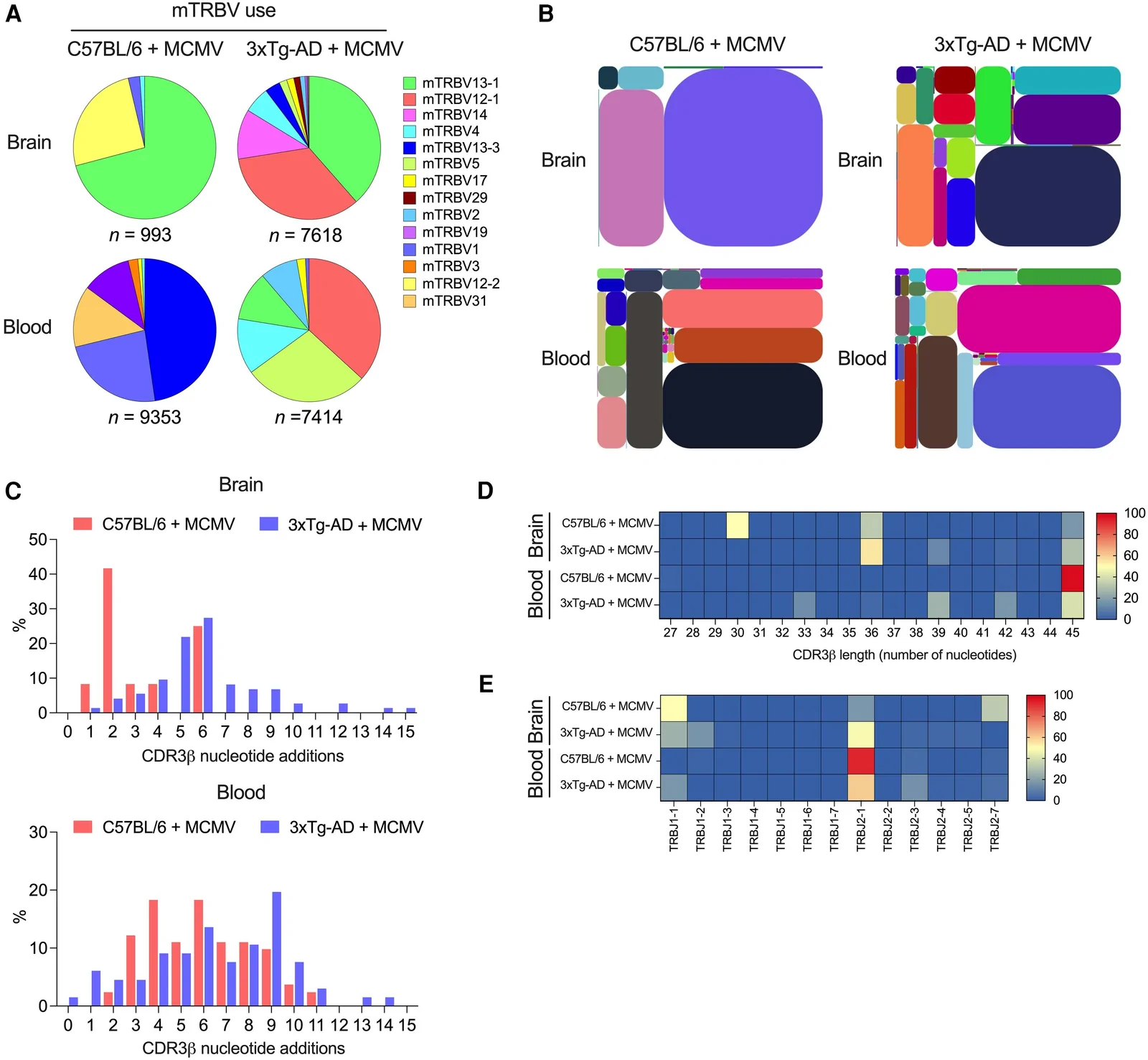
**MCMV-specific CD8^+^ T cells exhibit increased clonal diversity in 3xTg-AD mice.** C57BL/6 and 3xTg-AD mice were infected with 5 × 10^4^ pfu of MCMV (i.p.). Leucocytes were harvested from the blood and brains 6 months post-infection. (**A** and **B**) Concatenated frequencies of murine *TRBV* (mTRBV) gene transcripts (**A**) and tree maps of CDR3β clonotype use (**B**) in relation to the overall repertoire size of M38 tetramer^+^ CD8^+^ T cells from the blood and brains of C57BL/6 and 3xTg-AD mice. Different colours represent different T cell receptors (TCRs). Repertoire proportions are depicted by size. (**C**) TCR β-chain junctional nucleotide additions across the most prevalent M38 tetramer^+^ CD8^+^ T cell clonotypes from the blood and brains of C57BL/6 and 3xTg-AD mice. (**D** and **E**) Heat maps depicting the distributions of CDR3β length (**D**) and *TRBJ* gene use (**E**) among TCR β-chains expressed by M38 tetramer^+^ CD8^+^ T cells from the blood and brains of C57BL/6 and 3xTg-AD mice. All data are concatenated from pooled M38 tetramer^+^ CD8^+^ T cells sorted from the blood or brains of C57BL/6 or 3xTg-AD mice (*n* = 6 mice/group). AD = Alzheimer's disease; i.p. = intraperitoneal; MCMV = murine cytomegalovirus.

CD8^+^ T cells undergo antigen-driven proliferation in response to infection and often express structurally biased TCRs.^50^ To examine this phenomenon in MCMV-infected mice in relation to the development of AD, we sequenced TCR β-chain variable (*TRBV*) gene rearrangements expressed in CD8^+^ T cells specific for the H-2K^b^-restricted epitope from M38. Clonal diversity was markedly increased among M38-specific CD8^+^ T cells in brain tissue from MCMV-infected 3xTg-AD versus C57BL/6 mice (Fig. 5A and B and Supplementary Fig. 5A) but largely equivalent among M38-specific CD8^+^ T cells in blood from MCMV-infected 3xTg-AD versus C57BL/6 mice (Fig. 5A and B and Supplementary Fig. 5A and B). TCR clonotype overlap was also more pronounced between M38-specific CD8^+^ T cells in brain tissue and blood from 3xTg-AD versus C57BL/6 mice (Fig. 5A and B and Table 1). Moreover, greater numbers of junctional nucleotide additions, which shape repertoire diversity,^51^ were detected among the *TRBV* gene rearrangements expressed by M38-specific CD8^+^ T cells in brain tissue but not in blood from 3xTg-AD versus C57BL/6 mice (Fig. 5C). Concordant differences were noted in CDR3β length and *TRBJ* gene use (Fig. 5D and E).

**Table 1:** MCMV-specific CD8^+^ T cells exhibit increased clonal diversity in 3xTg-AD mice

mTRBV	CDR3β	mTRBJ	Frequency (%)
**C57BL/6 + MCMV (top 10 clonotypes) Brain**			
mTRBV13-1	CASSDWGTEVF	mTRBJ1-1	70.09
mTRBV12-2	CASSPGLGGYEQY	mTRBJ2-7	25.18
**mTRBV1**	**CTCSAGTGGYNYAEQF**	**mTRBJ2-1**	**2**.**62**
**mTRBV4**	**CASSPGTGGYNYAEQF**	**mTRBJ2-1**	**1**.**11**
mTRBV13-1	CASSDWGAEVF	mTRBJ1-1	0.50
mTRBV13-1	CASSGWGTEVF	mTRBJ1-1	0.30
mTRBV12-2	CASPPGLGGYEQY	mTRBJ2-7	0.20
**C57BL/6 + MCMV (top 10 clonotypes) Blood**			
mTRBV13-3	CASSPGTGGYNYAEQF	mTRBJ2-1	36.77
**mTRBV1**	**CTCSAGTGGYNYAEQF**	**mTRBJ2-1**	**16**.**55**
mTRBV31	CAWSPRTGGYNYAEQF	mTRBJ2-1	15.08
mTRBV13-3	CASSAGTGGYNYAEQF	mTRBJ2-1	13.94
mTRBV5	CASSQGTGGYNYAEQF	mTRBJ2-1	4.00
mTRBV1	CTCSGGTGGYNYAEQF	mTRBJ2-1	3.69
mTRBV1	CTCSAGTGGFNYAEQF	mTRBJ2-1	3.16
mTRBV5	CASSPGTGGFNYAEQF	mTRBJ2-1	2.35
mTRBV5	CASSQDRRARAETLY	mTRBJ2-3	2.26
mTRBV3	CASSPGTGGYNYAEQF	mTRBJ2-1	2.21
**3xTg-AD + MCMV (top 10 clonotypes) Brain**			
mTRBV13-1	CASSDGQGFTEVF	mTRBJ1-1	33.49
mTRBV12-1	CASSRGDNYAEQF	mTRBJ2-1	30.14
mTRBV14	CASSAGTGYSDYT	mTRBJ1-2	12.01
**mTRBV12-1**	**CASSPRTGGYNYAEQF**	**mTRBJ2-1**	**4**.**69**
mTRBV13-1	CASNFLGAANTEVF	mTRBJ1-1	4.18
mTRBV13-1	CASSLAASAETLY	mTRBJ2-3	4.09
mTRBV13-3	CASSPGTGGFNYAEQF	mTRBJ2-1	3.77
**mTRBV4**	**CASSPGTGGYNYAEQF**	**mTRBJ2-1**	**3**.**47**
mTRBV12-1	CASSSRTGGYNYAEQF	mTRBJ2-1	2.25
**mTRBV5**	**CASSQEGTYANTEVF**	**mTRBJ1-1**	**1**.**92**
**3xTg-AD + MCMV (top 10 clonotypes) Blood**			
** mTRBV12-1**	**CASSPRTGGYNYAEQF**	**mTRBJ2-1**	**39**.**96**
** mTRBV5**	**CASSQEGTYANTEVF**	**mTRBJ1-1**	**14**.**59**
mTRBV13-1	CASSGDYYAEQF	mTRBJ2-1	11.97
mTRBV5	CASSQEPNWGKTLY	mTRBJ2-3	8.17
mTRBV5	CASSPGTGGYNYAEQF	mTRBJ2-1	7.53
mTRBV4	CASSSGTGGYNYAEQF	mTRBJ2-1	5.29
mTRBV2	CASSQESWPYAEQF	mTRBJ2-1	3.46
** mTRBV4**	**CASSPGTGGYNYAEQF**	**mTRBJ2-1**	**3**.**13**
mTRBV2	CASSQEGWGGGYAEQF	mTRBJ2-1	3.05
mTRBV4	CASSAGTGGYNYAEQF	mTRBJ2-1	2.85

T cell receptor β-chain amino acid sequences expressed by M38 tetramer^+^ CD8^+^ T cells from the blood and brains of C57BL/6 and 3xTg-AD mice showing the top 10 most frequent clonotypes in each repertoire. Bold sequences are identical. AD = Alzheimer's disease; MCMV = murine cytomegalovirus.

These data are consistent with the notion that broad expansions of virus-specific CD8^+^ T cells, likely reflecting bilateral exchange with the vascular circulation, occur in the brains of MCMV-infected 3xTg-AD mice during the development of AD.

### T cells drive the development of AD induced by MCMV

**Figure 6:**
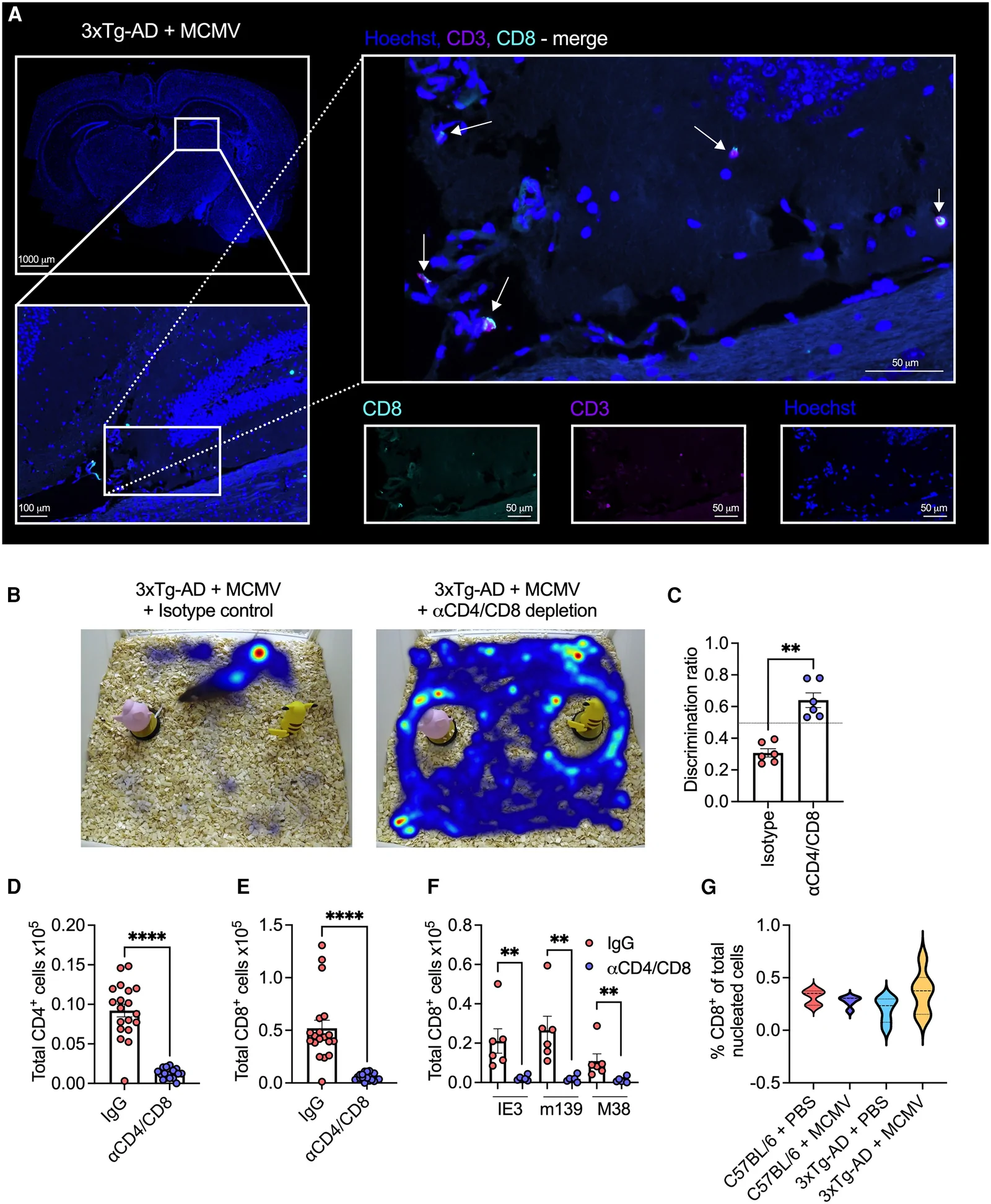
**T cells drive MCMV-induced cognitive decline in 3xTg-AD mice.** C57BL/6 and 3xTg-AD mice were mock-infected with PBS or infected with 5 × 10^4^ pfu of MCMV (i.p.). Mice were treated with depleting antibodies targeting CD4 and CD8 or an isotype control antibody 6 months post-infection. (**A**) Microscopy analysis of a coronal brain section (*top left*) showing CD3^+^ (purple)/CD8^+^ (cyan) T cells (white arrows) in the hippocampus (*bottom left* and *right*) of a 3xTg-AD mouse. Data are shown as merged expression (*top right*). Individual staining is shown for CD3, CD8 and Hoechst (*bottom right*). (**B** and **C**) Animal behaviour and cognitive performance were assessed using novel object recognition (NOR) heat maps (**B**) and post-NOR discrimination ratios (DRs) (**C**). Impaired cognition is represented by a DR below 0.5. (**D**–**F**) CD4^+^ T cells (**D**), CD8^+^ T cells (**E**) and MCMV tetramer^+^ CD8^+^ T cells (**F**) in the brains of 3xTg-AD mice quantified via flow cytometry. (**G**) Percentage frequencies of CD3^+^ (purple)/CD8^+^ (cyan) T cells from (**A**) among total nucleated cells (blue) in the hippocampi of C57BL/6 and 3xTg-AD mice. Data are shown as mean ± standard error of the mean. All data are representative of two independent experiments with *n* = 5–6 C57BL/6 and *n* = 5–6 3xTg-AD mice/group. Significance was assessed using the Mann–Whitney U-test. *P*-values are reported as follows: n.s. = *P* > 0.05, *P ≤ 0.05, **P ≤ 0.01, ***P ≤ 0.001, ****P ≤ 0.0001. AD = Alzheimer's disease; i.p. = intraperitoneal; MCMV = murine cytomegalovirus.

Histological analyses revealed that CD8^+^ T cells in the brains of MCMV-infected 3xTg-AD mice accumulated in the region of the hippocampus (Fig. 6A and G), the site of virus-associated neuronal damage (Fig. 2A and B). Moreover, we observed that concentrations of the cytokine interleukin (IL)-18, which can activate T cells in the absence of antigen,^52^ were higher in brain homogenates from MCMV-infected 3xTg-AD versus C57BL/6 mice (Supplementary Fig. 6). Improved cognitive function in MCMV-infected 3xTg-AD mice treated with valganciclovir was also associated with a reduction in brain-infiltrating CD8^+^ T cells specific for IE3 and M38 (Fig. 3B–D), suggesting a key role in the development of AD. To test this notion, we treated 3xTg-AD mice with anti-CD4 and anti-CD8 depleting antibodies during chronic MCMV infection. This intervention dramatically improved cognitive performance, as measured via NOR (Fig. 6B and C), and eliminated brain-infiltrating CD4^+^ and CD8^+^ T cells in MCMV-infected 3xTg-AD mice (Fig. 6D–F).

These findings provide direct evidence that MCMV-induced CD4^+^ and/or CD8^+^ T cells potentiate cognitive decline in a mouse model of AD.

## Discussion

Mechanistic studies are urgently required to understand, predict and potentially mitigate the risk of infectious exacerbations of AD. Here, we combined two well-established mouse models to investigate how infection with a representative herpesvirus, MCMV, could impact the development of AD. We found that MCMV triggered an influx of CD4^+^ and, to a greater extent, CD8^+^ T cells into the brains of 3xTg-AD mice, which contain three genetic mutations associated with familial AD. A vast majority of the brain-infiltrating CD8^+^ T cells exhibited a highly differentiated effector memory phenotype and recognized defined antigens from MCMV via clonally diverse TCRs. Brain tissues contained no detectable MCMV DNA. Virus-induced cognitive decline was ameliorated by administration of the antiviral drug valganciclovir and by antibody-mediated depletion of CD4^+^ and CD8^+^ T cells during chronic infection. These results provide direct evidence that a virus-induced immune response can accelerate the progression of AD.

It remains unclear how virus-induced T cells cause neuropathology in 3xTg-AD mice. One possibility is direct neuronal cytotoxicity. However, it seems unlikely that all brain-infiltrating CD8^+^ T cells would exhibit off-target specificity for neuronal self-antigens, especially given the observed clonal diversity and broad targeting of epitopes derived from MCMV. It is notable here that elevated concentrations of interferon (IFN)-γ have been observed in HCMV-seropositive patients with AD.^23^ IFN-γ is expressed by activated leucocytes and can inhibit dendrite outgrowth, which is associated with decreased rates of synapse formation.^53^ Importantly, neutralization of IFN-γ ameliorates pathology in a T cell-driven model of tauopathy^21^ and reduces plaque burden in APP/PS1 mice.^54^ These data suggest that virus-induced T cells promote disease progression via the expression of IFN-γ.

CD8^+^ T cells were detectable in the hippocampus, a site of infection-induced neuronal damage, and dominated the overall leucocyte infiltrate in the brains of MCMV-infected 3xTg-AD mice. Similarly, CD8^+^ T cells have been reported in the brains of patients with AD,^17,19^ and EBV-specific CD8^+^ T cells have been detected in the CSF of patients with AD.^18^ It nonetheless remains possible that CD4^+^ T cells also play a role in disease progression. Indeed, HLA-DRB1 is a risk locus for AD,^55^ and CD4^+^ Th1 and Th17 cells exhibit inflammatory functionality in experimental models of AD.^54,56^ Additional work is required to disentangle the relative contributions of CD4^+^ versus CD8^+^ T cells in the pathogenesis of infection-induced AD.

3xTg-AD mice exhibit splenomegaly and lymphoid cell expansions in the periphery.^35^ We also observed high frequencies of virus-specific CD8^+^ T cells in lung tissue during chronic MCMV infection. However, we found no evidence of a role for peripheral immune dysregulation in the pathogenesis of MCMV-induced AD, given that the clonal architecture and phenotypic characteristics of circulating virus-specific CD8^+^ T cells were similar in C57BL/6 and 3xTg-AD mice. CD4^−^CD8^−^ T cells dominate the peripheral perturbations observed in 3xTg-AD mice.^35,57^ In contrast we found that antibody-mediated depletion of CD4^+^ and CD8^+^ T cells ameliorated disease progression consistent with a role for these T cell subsets and not CD4^−^CD8^−^ T cells in disease development. It is important to note however, that we did not deplete each subset separately to dissect lineage-specific contributions to the pathogenesis of MCMV-induced AD.

Valganciclovir treatment during the chronic phase of infection alleviated cognitive decline and reversed the associated influx of MCMV-specific CD8^+^ T cells into the brains of 3xTg-AD mice. This latter observation could be explained by the suppression of viral reactivation. However, it has also been suggested that MCMV-induced CD8^+^ T cell expansions during latency result from stochastic episodes of gene expression rather than productive viral reactivation,^58^ which is more difficult to integrate into a simple model of valganciclovir-induced antigen elimination. One possible explanation here is that localized proinflammatory environments preferentially drive viral reactivation in 3xTg-AD mice.^59^ Another possible but perhaps less likely explanation is that valganciclovir exerts cytotoxic effects on the immune system.

MCMV DNA was not detected in the brains of 3xTg-AD mice. Although we did not apply highly sensitive methods to quantify viral loads in specific regions of the brain, such as the hippocampus, our data suggest that lung and spleen tissue are the primary reservoirs of latent MCMV. This observation raises a key question. If the virus is absent from brain tissue, how are virus-specific CD4^+^ and CD8^+^ T cells recruited to the site of pathology in 3xTg-AD mice?

Repeat MCMV infection has been demonstrated to limit blood–brain barrier (BBB) integrity and cognition after repeated infectious challenges *in vivo.*^60^ Consistent with the established role for systemic infections in impacting BBB integrity,^61^ a single dose of MCMV can also increase BBB permeability, at least transiently.^60^ It therefore seems likely that increased BBB permeability in MCMV-infected 3xTg-AD mice contributes to T cell accumulations in brain tissue. However, the preferential enrichment of virus-specific effector memory CD8^+^ T cells suggests that a selective mechanism, potentially involving inflammatory chemokines,^49^ governs immune cell recruitment into the brains of 3xTg-AD mice. This possibility is supported by our finding that brain-infiltrating MCMV-specific CD8^+^ T cells were enriched for expression of the CXCL10 receptor CXCR3.^62^ Accordingly, virus-specific CD8^+^ T cells likely upregulate CXCR3 in response to antigen recognition in the periphery, enabling recruitment via the expression of CXCL10 in the brains of 3xTg-AD mice.^63^

Of note, we also found that IL-18 was upregulated in the brains of MCMV-infected 3xTg-AD mice, akin to a previous study of patients with AD.^64^ This cytokine can activate T cells in the absence of antigen^52^ and promote T cell proliferation.^65^ These observations suggest that CD4^+^ and CD8^+^ T cells could become activated to proliferate locally in brain tissue and produce cytokines and cytotoxins that contribute to the neuropathology of AD. Remarkably, at least 45 different pathogens, only some of which are neurotropic, have been associated with the development of AD.^6^ These data suggest that infectious agents may operate via a universal mechanism to exacerbate the neuropathology of AD.^16,66^ Our findings provide initial evidence linking cellular immune responses with infection-associated brain pathology, highlighting a key role for virus-specific CD4^+^ and CD8^+^ T cells in the accelerated development of AD.

## Supplementary Material

awag043_Supplementary_Data

## Data Availability

All original TCR sequencing data are freely available in fully annotated form via Zenodo (10.5281/zenodo.15125167).

## References

[1] Breijyeh Z, Karaman R. Comprehensive review on Alzheimer's disease: Causes and treatment. Molecules. 2020;25:5789. 33302541 10.3390/molecules25245789 PMC7764106

[2] Sims R, Hill M, Williams J. The multiplex model of the genetics of Alzheimer's disease. Nat Neurosci. 2020;23:311–322. 32112059 10.1038/s41593-020-0599-5

[3] Fischer OL . Miliare Nekrosen mit drusigen Wucherungen der Neurofibrillen, eine regelmässige Veränderung der Hirnrinde bei seniler Demenz. Monatsschr Psychiatr Neurol. 1907;22:361–372.

[4] Alzheimer A . Über eigenartige Krankheitsfälle des späteren Alters. Neur u Psych. 1911;4:356–385.

[5] Vojtechova I, Machacek T, Kristofikova Z, Stuchlik A, Petrasek T. Infectious origin of Alzheimer's disease: Amyloid beta as a component of brain antimicrobial immunity. PLoS Pathog. 2022;18:e1010929. 36395147 10.1371/journal.ppat.1010929 PMC9671327

[6] Levine KS, Leonard HL, Blauwendraat C, et al Virus exposure and neurodegenerative disease risk across national biobanks. Neuron. 2023;111:1086–1093.e2. 36669485 10.1016/j.neuron.2022.12.029 PMC10079561

[7] Wang L, Davis PB, Volkow ND, Berger NA, Kaelber DC, Xu R. Association of COVID-19 with new-onset Alzheimer's disease. J Alzheimers Dis. 2022;89:411–414. 35912749 10.3233/JAD-220717 PMC10361652

[8] Wozniak MA, Mee AP, Itzhaki RF. Herpes simplex virus type 1 DNA is located within Alzheimer's disease amyloid plaques. J Pathol. 2009;217:131–138. 18973185 10.1002/path.2449

[9] Jamieson GA, Maitland NJ, Wilcock GK, Yates CM, Itzhaki RF. *Herpes simplex* virus type 1 DNA is present in specific regions of brain from aged people with and without senile dementia of the Alzheimer type. J Pathol. 1992;167:365–368. 1328575 10.1002/path.1711670403

[10] Readhead B, Haure-Mirande J-V, Funk CC, et al Multiscale analysis of independent Alzheimer's cohorts finds disruption of molecular, genetic, and clinical networks by human herpesvirus. Neuron. 2018;99:64–82.e7. 29937276 10.1016/j.neuron.2018.05.023 PMC6551233

[11] Taquet M, Dercon Q, Todd JA, Harrison PJ. The recombinant shingles vaccine is associated with lower risk of dementia. Nat Med. 2024;30:2777–2781. 39053634 10.1038/s41591-024-03201-5 PMC11485228

[12] Barnes LL, Capuano AW, Aiello AE, et al Cytomegalovirus infection and risk of Alzheimer disease in older black and white individuals. J Infect Dis. 2015;211:230–237. 25108028 10.1093/infdis/jiu437 PMC4326304

[13] Aiello AE, Haan MN, Blythe L, Moore K, Gonzalez JM, Jagust W. The influence of latent viral infection on rate of cognitive decline over 4 years. J Am Geriatr Soc. 2006;54:1046–1054. 16866674 10.1111/j.1532-5415.2006.00796.x

[14] Readhead BP, Mastroeni DF, Wang Q, et al Alzheimer's disease-associated CD83^(+)^ microglia are linked with increased immunoglobulin G4 and human cytomegalovirus in the gut, vagal nerve, and brain. Alzheimers Dement. 2025;21:e14401. 39698934 10.1002/alz.14401 PMC11772737

[15] Wennberg AM, Maher BS, Rabinowitz JA, et al Association of common infections with cognitive performance in the Baltimore epidemiologic catchment area study follow-up. Alzheimers Dement. 2023;19:4841–4851. 37027458 10.1002/alz.13070 PMC10558626

[16] Clement M . The association of microbial infection and adaptive immune cell activation in Alzheimer's disease. Discov Immunol. 2023;2:kyad015. 38567070 10.1093/discim/kyad015 PMC10917186

[17] Merlini M, Kirabali T, Kulic L, Nitsch RM, Ferretti MT. Extravascular CD3^+^ T cells in brains of Alzheimer disease patients correlate with tau but not with amyloid pathology: An immunohistochemical study. Neurodegener Dis. 2018;18:49–56. 29402847 10.1159/000486200

[18] Gate D, Saligrama N, Leventhal O, et al Clonally expanded CD8 T cells patrol the cerebrospinal fluid in Alzheimer's disease. Nature. 2020;577:399–404. 31915375 10.1038/s41586-019-1895-7 PMC7445078

[19] Unger MS, Li E, Scharnagl L, et al CD8^(+)^ T-cells infiltrate Alzheimer's disease brains and regulate neuronal- and synapse-related gene expression in APP-PS1 transgenic mice. Brain Behav Immun. 2020;89:67–86. 32479993 10.1016/j.bbi.2020.05.070

[20] Lee SH, Rezzonico MG, Friedman BA, et al TREM2-independent oligodendrocyte, astrocyte, and T cell responses to tau and amyloid pathology in mouse models of Alzheimer disease. Cell Rep. 2021;37:110158. 34965428 10.1016/j.celrep.2021.110158

[21] Chen X, Firulyova M, Manis M, et al Microglia-mediated T cell infiltration drives neurodegeneration in tauopathy. Nature. 2023;615:668–677. 36890231 10.1038/s41586-023-05788-0 PMC10258627

[22] Dai L, Shen Y. Insights into T-cell dysfunction in Alzheimer's disease. Aging Cell. 2021;20:e13511. 34725916 10.1111/acel.13511 PMC8672785

[23] Lurain NS, Hanson BA, Martinson J, et al Virological and immunological characteristics of human cytomegalovirus infection associated with Alzheimer disease. J Infect Dis. 2013;208:564–572. 23661800 10.1093/infdis/jit210 PMC3719902

[24] Westman G, Berglund D, Widén J, et al Increased inflammatory response in cytomegalovirus seropositive patients with Alzheimer's disease. PLoS One. 2014;9:e96779. 24804776 10.1371/journal.pone.0096779 PMC4013077

[25] Oddo S, Caccamo A, Shepherd JD, et al Triple-transgenic model of Alzheimer's disease with plaques and tangles: Intracellular Aβ and synaptic dysfunction. Neuron. 2003;39:409–421. 12895417 10.1016/s0896-6273(03)00434-3

[26] Webberley TS, Bevan RJ, Kerry-Smith J, et al Assessment of Lab4P probiotic effects on cognition in 3xTg-AD Alzheimer's disease model mice and the SH-SY5Y neuronal cell line. Int J Mol Sci. 2023;24:4683. 36902113 10.3390/ijms24054683 PMC10003662

[27] Deacon RM, Rawlins JN. T-maze alternation in the rodent. Nat Protoc. 2006;1:7–12. 17406205 10.1038/nprot.2006.2

[28] Stack G, Jones E, Marsden M, et al CD200 receptor restriction of myeloid cell responses antagonizes antiviral immunity and facilitates cytomegalovirus persistence within mucosal tissue. PLoS Pathog. 2015;11:e1004641. 25654642 10.1371/journal.ppat.1004641 PMC4412112

[29] Clement M, Marsden M, Stacey MA, et al Cytomegalovirus-specific IL-10-producing CD4^+^ T cells are governed by type-I IFN-induced IL-27 and promote virus persistence. PLoS Pathog. 2016;12:e1006050. 27926930 10.1371/journal.ppat.1006050 PMC5142785

[30] Kamimura Y, Lanier LL. Rapid and sequential quantitation of salivary gland-associated mouse cytomegalovirus in oral lavage. J Virol Methods. 2014;205:53–56. 24747009 10.1016/j.jviromet.2014.03.029 PMC4201903

[31] Clement M, Ladell K, Miners KL, et al Inhibitory IL-10-producing CD4^(+)^ T cells are T-bet-dependent and facilitate cytomegalovirus persistence via coexpression of arginase-1. Elife. 2023;12:e79165. 37440306 10.7554/eLife.79165 PMC10344424

[32] Bevan RJ, Cimaglia G, Morgan JE, Taylor PR. Improved DiOlistic labelling technique for neurons in situ: Detailed visualisation of dendritic spines and concurrent histochemical-detection in fixed tissue. Methods. 2024;229:82–93. 38917961 10.1016/j.ymeth.2024.06.009

[33] Bankhead P, Loughrey MB, Fernández JA, et al Qupath: Open source software for digital pathology image analysis. Sci Rep. 2017;7:16878. 29203879 10.1038/s41598-017-17204-5 PMC5715110

[34] Pairojana T, Phasuk S, Suresh P, et al Age and gender differences for the behavioral phenotypes of 3xTg Alzheimer's disease mice. Brain Res. 2021;1762:147437. 33753066 10.1016/j.brainres.2021.147437

[35] Nava Catorce M, Acero G, Gevorkian G. Age- and sex-dependent alterations in the peripheral immune system in the 3xTg-AD mouse model of Alzheimer's disease: Increased proportion of CD3^+^CD4^-^CD8^-^ double-negative T cells in the blood. J Neuroimmunol. 2021;360:577720. 34543880 10.1016/j.jneuroim.2021.577720

[36] Makrigiannis AP, Pau AT, Saleh A, Winkler-Pickett R, Ortaldo JR, Anderson SK. Class I MHC-binding characteristics of the 129/J Ly49 repertoire. J Immunol. 2001;166:5034–5043. 11290784 10.4049/jimmunol.166.8.5034

[37] Scalzo AA, Fitzgerald NA, Simmons A, La Vista AB, Shellam GR. Cmv-1, a genetic locus that controls murine cytomegalovirus replication in the spleen. J Exp Med. 1990;171:1469–1483. 2159050 10.1084/jem.171.5.1469 PMC2187882

[38] Reuter JD, Gomez DL, Wilson JH, van den Pol AN. Systemic immune deficiency necessary for cytomegalovirus invasion of the mature brain. J Virol. 2004;78:1473–1487. 14722303 10.1128/JVI.78.3.1473-1487.2004 PMC321365

[39] Krstanović F, Mihalić A, Šakota L, Lisnić B, Jonjić S, Brizić I. Susceptibility of mouse brain to MCMV infection and neuroinflammation during ontogeny. Pathogens. 2024;13:1108. 39770367 10.3390/pathogens13121108 PMC11728524

[40] Beswick M, Pachnio A, Lauder SN, Sweet C, Moss PA. Antiviral therapy can reverse the development of immune senescence in elderly mice with latent cytomegalovirus infection. J Virol. 2013;87:779–789. 23115277 10.1128/JVI.02427-12 PMC3554082

[41] Pchitskaya E, Bezprozvanny I. Dendritic spines shape analysis-classification or clusterization? Perspective. Front Synaptic Neurosci. 2020;12:31. 33117142 10.3389/fnsyn.2020.00031 PMC7561369

[42] Helm MS, Dankovich TM, Mandad S, et al A large-scale nanoscopy and biochemistry analysis of postsynaptic dendritic spines. Nat Neurosci. 2021;24:1151–1162. 34168338 10.1038/s41593-021-00874-w

[43] Javonillo DI, Tran KM, Phan J, et al Systematic phenotyping and characterization of the 3xTg-AD mouse model of Alzheimer's disease. Front Neurosci. 2021;15:785276. 35140584 10.3389/fnins.2021.785276 PMC8818877

[44] Mody PH, Marvin KN, Hynds DL, Hanson LK. Cytomegalovirus infection induces Alzheimer's disease-associated alterations in tau. J Neurovirol. 2023;29:400–415. 37436577 10.1007/s13365-022-01109-9

[45] Munks MW, Gold MC, Zajac AL, et al Genome-wide analysis reveals a highly diverse CD8 T cell response to murine cytomegalovirus. J Immunol. 2006;176:3760–3766. 16517745 10.4049/jimmunol.176.6.3760

[46] Arens R, Wang P, Sidney J, et al Cutting edge: Murine cytomegalovirus induces a polyfunctional CD4 T cell response. J Immunol. 2008;180:6472–6476. 18453564 10.4049/jimmunol.180.10.6472 PMC2587066

[47] Walton SM, Wyrsch P, Munks MW, et al The dynamics of mouse cytomegalovirus-specific CD4 T cell responses during acute and latent infection. J Immunol. 2008;181:1128–1134. 18606665 10.4049/jimmunol.181.2.1128

[48] Munks MW, Cho KS, Pinto AK, Sierro S, Klenerman P, Hill AB. Four distinct patterns of memory CD8 T cell responses to chronic murine cytomegalovirus infection. J Immunol. 2006;177:450–458. 16785542 10.4049/jimmunol.177.1.450

[49] Wojcieszak J, Kuczyńska K, Zawilska JB. Role of chemokines in the development and progression of Alzheimer's disease. J Mol Neurosci. 2022;72:1929–1951. 35821178 10.1007/s12031-022-02047-1 PMC9392685

[50] Turner SJ, Doherty PC, McCluskey J, Rossjohn J. Structural determinants of T-cell receptor bias in immunity. Nat Rev Immunol. 2006;6:883–894. 17110956 10.1038/nri1977

[51] Venturi V, Kedzierska K, Price DA, et al Sharing of T cell receptors in antigen-specific responses is driven by convergent recombination. Proc Natl Acad Sci U S A. 2006;103:18691–18696. 17130450 10.1073/pnas.0608907103 PMC1693724

[52] Berg RE, Crossley E, Murray S, Forman J. Memory CD8^+^ T cells provide innate immune protection against *Listeria monocytogenes* in the absence of cognate antigen. J Exp Med. 2003;198:1583–1593. 14623912 10.1084/jem.20031051 PMC1592647

[53] Kim IJ, Beck HN, Lein PJ, Higgins D. Interferon-γ induces retrograde dendritic retraction and inhibits synapse formation. J Neurosci. 2002;22:4530–4539. 12040060 10.1523/JNEUROSCI.22-11-04530.2002 PMC6758811

[54] Browne TC, McQuillan K, McManus RM, O’Reilly J-A, Mills KHG, Lynch MA. IFN-γ production by amyloid β-specific Th1 cells promotes microglial activation and increases plaque burden in a mouse model of Alzheimer's disease. J Immunol. 2013;190:2241–2251. 23365075 10.4049/jimmunol.1200947

[55] Kunkle BW, Grenier-Boley B, Sims R, et al Author correction: Genetic meta-analysis of diagnosed Alzheimer's disease identifies new risk loci and implicates Aβ, tau, immunity and lipid processing. Nat Genet. 2019;51:1423–1424. 31417202 10.1038/s41588-019-0495-7 PMC7265117

[56] Machhi J, Yeapuri P, Lu Y, et al CD4^+^ effector T cells accelerate Alzheimer's disease in mice. J Neuroinflammation. 2021;18:272. 34798897 10.1186/s12974-021-02308-7 PMC8603581

[57] Marchese M, Cowan D, Head E, et al Autoimmune manifestations in the 3xTg-AD model of Alzheimer's disease. J Alzheimers Dis. 2014;39:191–210. 24150111 10.3233/JAD-131490 PMC4376359

[58] Griessl M, Renzaho A, Freitag K, Seckert CK, Reddehase MJ, Lemmermann NAW. Stochastic episodes of latent cytomegalovirus transcription drive CD8 T-cell “memory inflation” and avoid immune evasion. Front Immunol. 2021;12:668885. 33968074 10.3389/fimmu.2021.668885 PMC8100209

[59] Cook CH, Trgovcich J, Zimmerman PD, Zhang Y, Sedmak DD. Lipopolysaccharide, tumor necrosis factor alpha, or interleukin-1β triggers reactivation of latent cytomegalovirus in immunocompetent mice. J Virol. 2006;80:9151–9158. 16940526 10.1128/JVI.00216-06 PMC1563908

[60] Harrison MAA, Morris SL, Rudman GA, et al Intermittent cytomegalovirus infection alters neurobiological metabolism and induces cognitive deficits in mice. Brain Behav Immun. 2024;117:36–50. 38182037 10.1016/j.bbi.2023.12.033 PMC11914963

[61] Galea I . The blood-brain barrier in systemic infection and inflammation. Cell Mol Immunol. 2021;18:2489–2501. 34594000 10.1038/s41423-021-00757-x PMC8481764

[62] Groom JR, Luster AD. CXCR3 in T cell function. Exp Cell Res. 2011;317:620–631. 21376175 10.1016/j.yexcr.2010.12.017 PMC3065205

[63] Zaheer S, Thangavel R, Wu Y, Khan MM, Kempuraj D, Zaheer A. Enhanced expression of glia maturation factor correlates with glial activation in the brain of triple transgenic Alzheimer's disease mice. Neurochem Res. 2013;38:218–225. 23086473 10.1007/s11064-012-0913-z PMC3527678

[64] Ojala J, Alafuzoff I, Herukka S-K, van Groen T, Tanila H, Pirttilä T. Expression of interleukin-18 is increased in the brains of Alzheimer's disease patients. Neurobiol Aging. 2009;30:198–209. 17658666 10.1016/j.neurobiolaging.2007.06.006

[65] Iwai Y, Hemmi H, Mizenina O, Kuroda S, Suda K, Steinman RM. An IFN-γ-IL-18 signaling loop accelerates memory CD8^+^ T cell proliferation. PLoS One. 2008;3:e2404. 18545704 10.1371/journal.pone.0002404 PMC2408965

[66] Hosseininasab SSM, Dadkhah P, Alizadeh Ojoor A, et al Alzheimer's disease and infectious agents: A comprehensive review of pathogenic mechanisms and microRNA roles. Front Neurosci. 2024;18:1513095. 39840010 10.3389/fnins.2024.1513095 PMC11747386

[67] Percie du Sert N, Hurst V, Ahluwalia A, et al The ARRIVE guidelines 2.0: Updated guidelines for reporting animal research. BMJ Open Sci. 2020;4:e100115. 10.1136/bmjos-2020-100115 PMC7610906 34095516

