# Supplementary material for: Cytomegalovirus-induced T cell responses accelerate Alzheimer’s disease progression in mice
Source: Brain. 2026 Jul 13;149(9):3214–27. doi: 10.1093/brain/awag043 (PMC13548865; doi:10.1093/brain/awag043)

SUPPLEMENTAL FIGURE LEGENDS

Figure S1. 3xTg-AD mice control MCMV similarly to C57BL/6 mice. C57BL/6, B6129SF2/J, and 3xTg-AD mice were infected with 3×10^4 pfu of MCMV (i.p.). (**A**, **B**, **E**) Survival (**A**), weight loss (**B**), and viral genomes measured in saliva via qPCR at d35 post-infection (**E**). The survival curves overlap for C57BL/6 and 3xTg-AD mice. (**C&D**) Replicating virus was measured via plaque assay in liver, lung, and spleen homogenates at d4 post-infection (**C**) and in liver and salivary gland homogenates at d14 post-infection (**D**). Data are shown as mean ± SEM. Data are representative of 2 independent experiments with n = 6 C57BL/6, n = 6 B6129SF2/J, and n = 6 3xTg-AD mice/group (**B–D**) or n = 8 C57BL/6 and n = 9 3xTg-AD mice/group (**E**). Significance was assessed using the Mantel–Cox test (**A**), the Mann–Whitney U test (**B**), or a one-way ANOVA with Tukey's post-hoc correction on LOG transformed data (**C&D**). p values are reported as follows: n.s., >0.05; *, ≤0.05; **, ≤0.01; ***, ≤0.001; and ****, ≤0.0001.

Figure S2. 3xTg-AD mice elicit NK cell responses comparable to C57BL/6 but not B6129SF2/J mice. (**A&B**) C57BL/6, B6129SF2/J, and 3xTg-AD mice were mock-infected with PBS (**A**) or infected with 3×10^4 pfu of MCMV (i.p.) (**B**). The graphs show percentage frequencies of $CD3^{-}NK1.1^{+}NKp46^{+}Ly49H^{+}$ cells, total numbers of $CD3^{-}NK1.1^{+}NKp46^{+}Ly49H^{+}$ cells, and total numbers of $CD3^{-}NK1.1^{+}NKp46^{+}$ cells quantified via flow cytometry. Data are shown as mean ± SEM. Data are representative of 1 independent experiment with n = 6 C57BL/6, n = 6 B6129SF2/J, and n = 6 3xTg-AD mice/group. Significance was assessed using a two-way ANOVA with Tukey's post-hoc correction. p values are reported as follows: n.s., >0.05; *, ≤0.05; **, ≤0.01; ***, ≤0.001; and ****, ≤0.0001.

Figure S3. Measurable viral latency is not established in the brains of 3xTg-AD mice. (**A–C**) C57BL/6, B6129SF2/J, and 3xTg-AD mice were mock-infected with PBS or infected with 3×10^4 pfu of MCMV (i.p.). DNA genome copy number was measured in the brains (cortex), lungs, and spleens via qPCR. Where viral load is undetectable an arbitrary CT value of 40 (max cycle number) was used. Data are shown as individual mice + mean. Data are representative of

1 independent experiment with *n* = 6 C57BL/6, *n* = 6 B6129SF2/J, and *n* = 6 3xTg-AD mice/group.

Figure S4. Characteristics of brain-infiltrating and circulating $CD8^+$ T cells in MCMV-infected C57BL/6 and 3xTg-AD mice. C57BL/6 and 3xTg-AD mice were mock-infected with PBS or infected with 5×10^4 pfu of MCMV (**A&B, D–F**) or 3×10^4 pfu of MCMV (i.p.) (**C**). Some groups of mice were treated with 1mg/ml valganciclovir hydrochloride dissolved in animal drinking water continuously from d60 post-infection. (**A**) Numbers of $CD44^-CD62L^+CD127^+$ $CD4^+$ and $CD8^+$ T cells in the brains of 3xTg-AD mice. (**B**) Percentage frequencies of $CD11a^+CD69^+$ $CD4^+$ T_{RM} cells (left) and $CD69^+CD103^+$ $CD8^+$ T_{RM} cells (right) in the brains of C57BL/6 and 3xTg-AD mice. (**C**) Paired analyses of CX3CR1 and CXCR3 expression among m139 $tetramer^+$ (left) and M45 $tetramer^+$ $CD8^+$ T cells (right) in the blood versus brains of 3xTg-AD mice. Significance was assessed using Wilcoxon matched pairs test. p values are reported as follows: n.s., >0.05; *, ≤0.05; **, ≤0.01; ***, ≤0.001; and ****, ≤0.0001. (**D**–**F**) Percentage frequencies of $CD8^+$ T cells (**D**), IE3 $tetramer^+$ $CD8^+$ T cells (E), and M38 $tetramer^+$ $CD8^+$ T cells (**F**) in the blood of C57BL/6 and 3xTg-AD mice over 180 days after infection with MCMV. Data are representative of 2 independent experiments with *n* = 8 3xTg-AD mice/group (**A**), *n* = 4 C57BL/6 and *n* = 9–10 3xTg-AD mice/group (**B&D**), or *n* = 6 3xTg-AD mice/group (**C**).

Figure S5. MCMV-specific $CD8^+$ T cells exhibit increased clonally diversity in 3xTg-AD mice. C57BL/6 and 3xTg-AD mice were infected with 5×10^4 pfu of MCMV (i.p.). Leukocytes were harvested from the blood and brains 6 months post-infection. (**A&B**) Concatenated clonotype frequencies showing murine *TRBV* (mTRBV) gene transcripts (**A**) and the normalized Shannon–Wiener index (**B**) reflecting clonality in relation to the overall repertoire size of M38 $tetramer^+$ $CD8^+$ T cells from the blood and brains of C57BL/6 and 3xTg-AD mice. All data are concatenated from pooled M38 $tetramer^+$ $CD8^+$ T cells sorted from the blood or brains of C57BL/6 or 3xTg-AD mice (*n* = 6 mice/group).

Figure S6. MCMV infection drives proinflammatory cytokine release in the brains of 3xTg-AD mice. C57BL/6 and 3xTg-AD mice were mock-infected with PBS or infected with 5×10^4 pfu of MCMV (i.p.). IL-18 concentrations in brain homogenates were measured 6 months post-infection via ELISA. Data are representative of *n* = 3–4 C57BL/6 and *n* = 10

3xTg-AD mice/group. Significance was assessed using a one-way ANOVA with Tukey's post-hoc correction. p values are reported as follows: n.s., >0.05; *, ≤0.05; **, ≤0.01; ***, ≤0.001; and ****, ≤0.0001.

Supplemental Figure 1

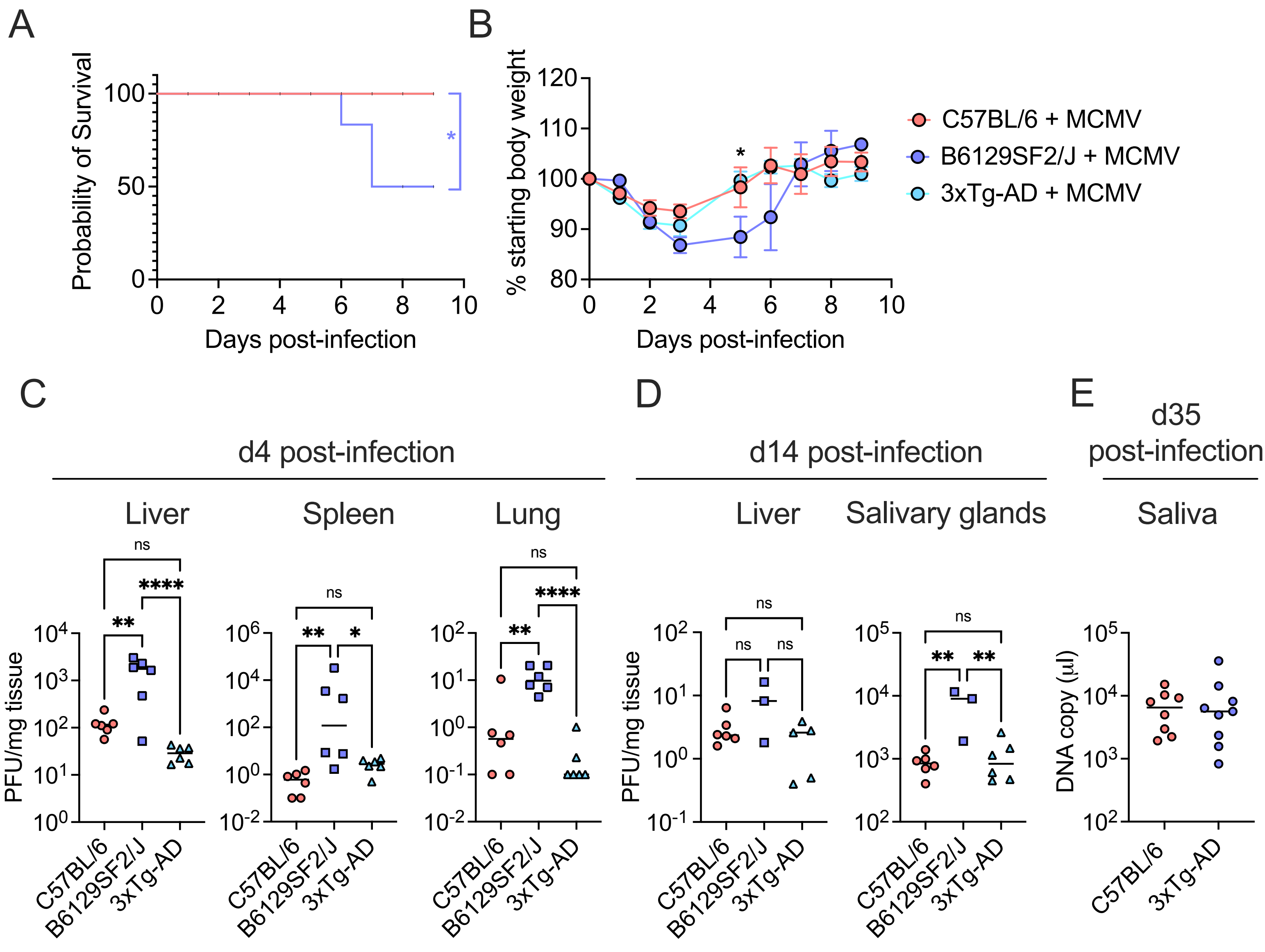

Supplemental Figure 2

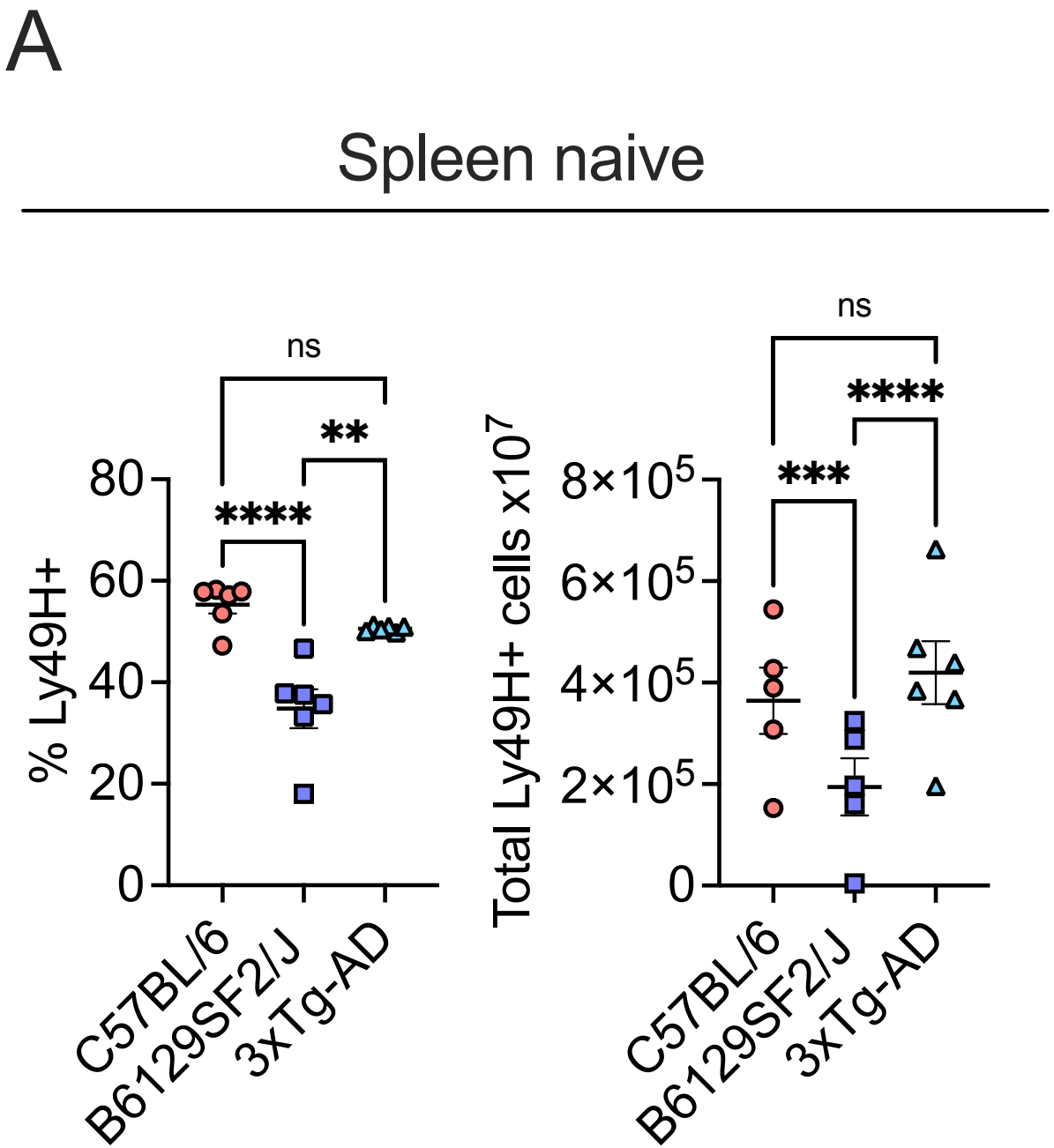

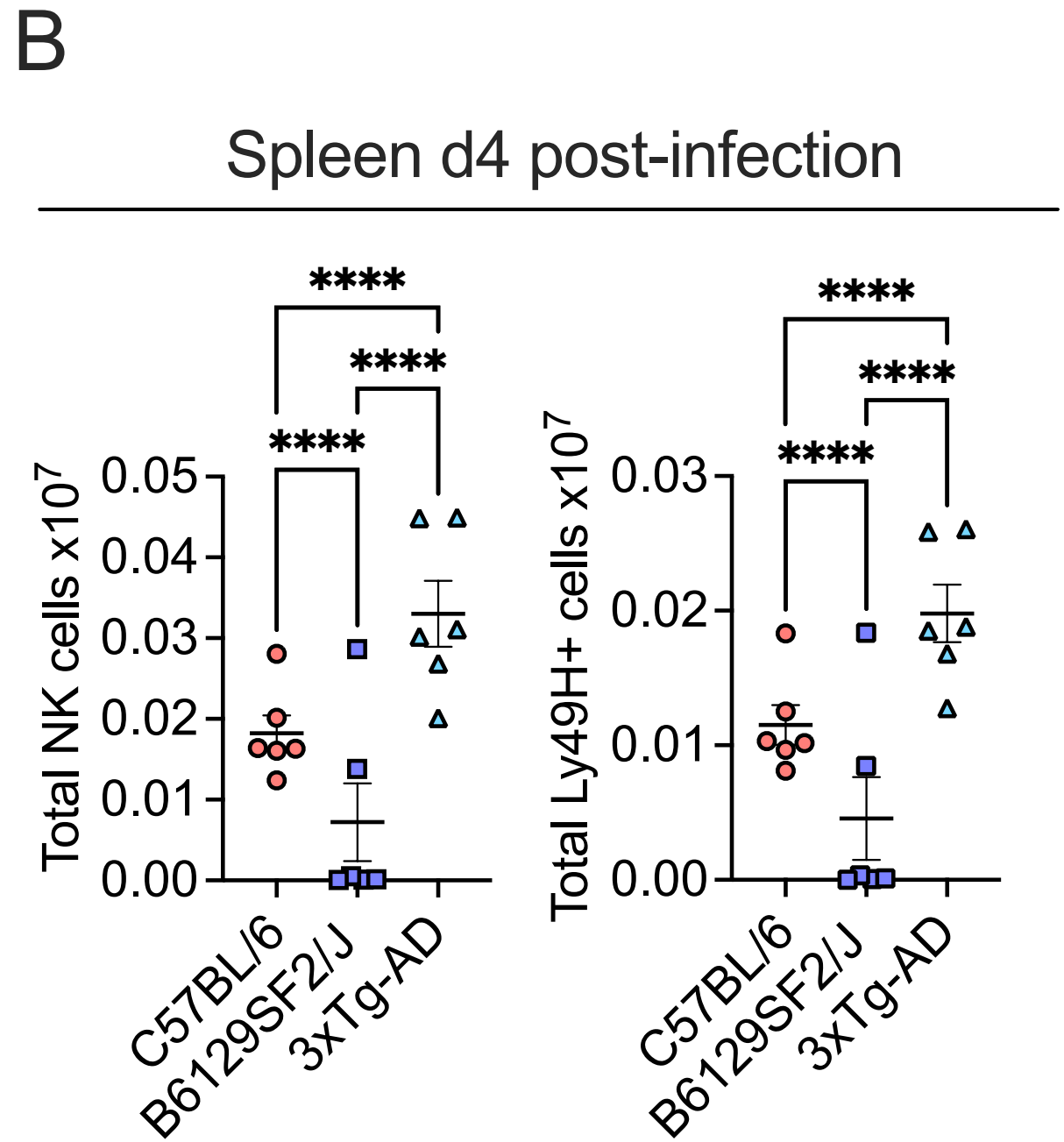

Supplemental Figure 3

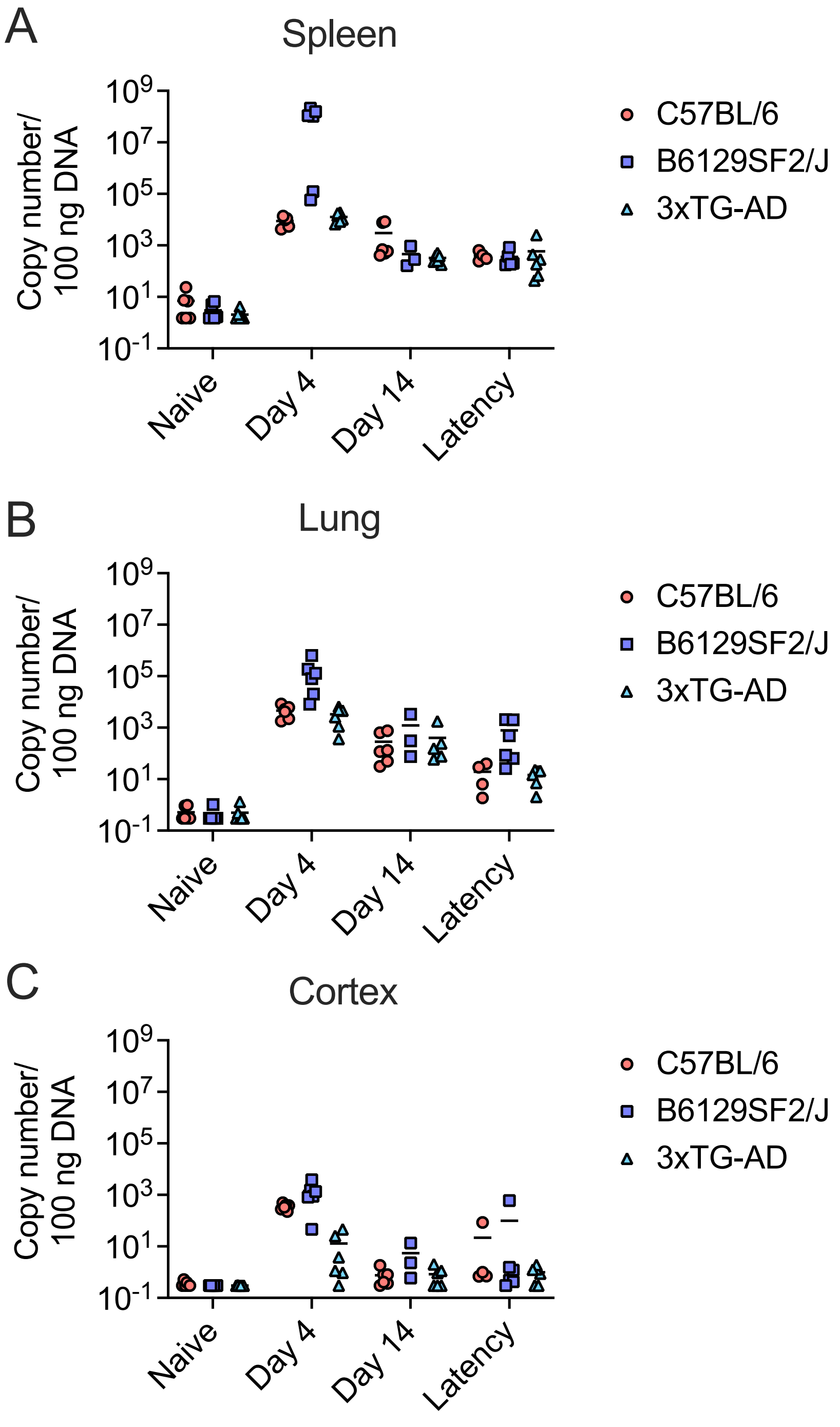

Supplemental Figure 4

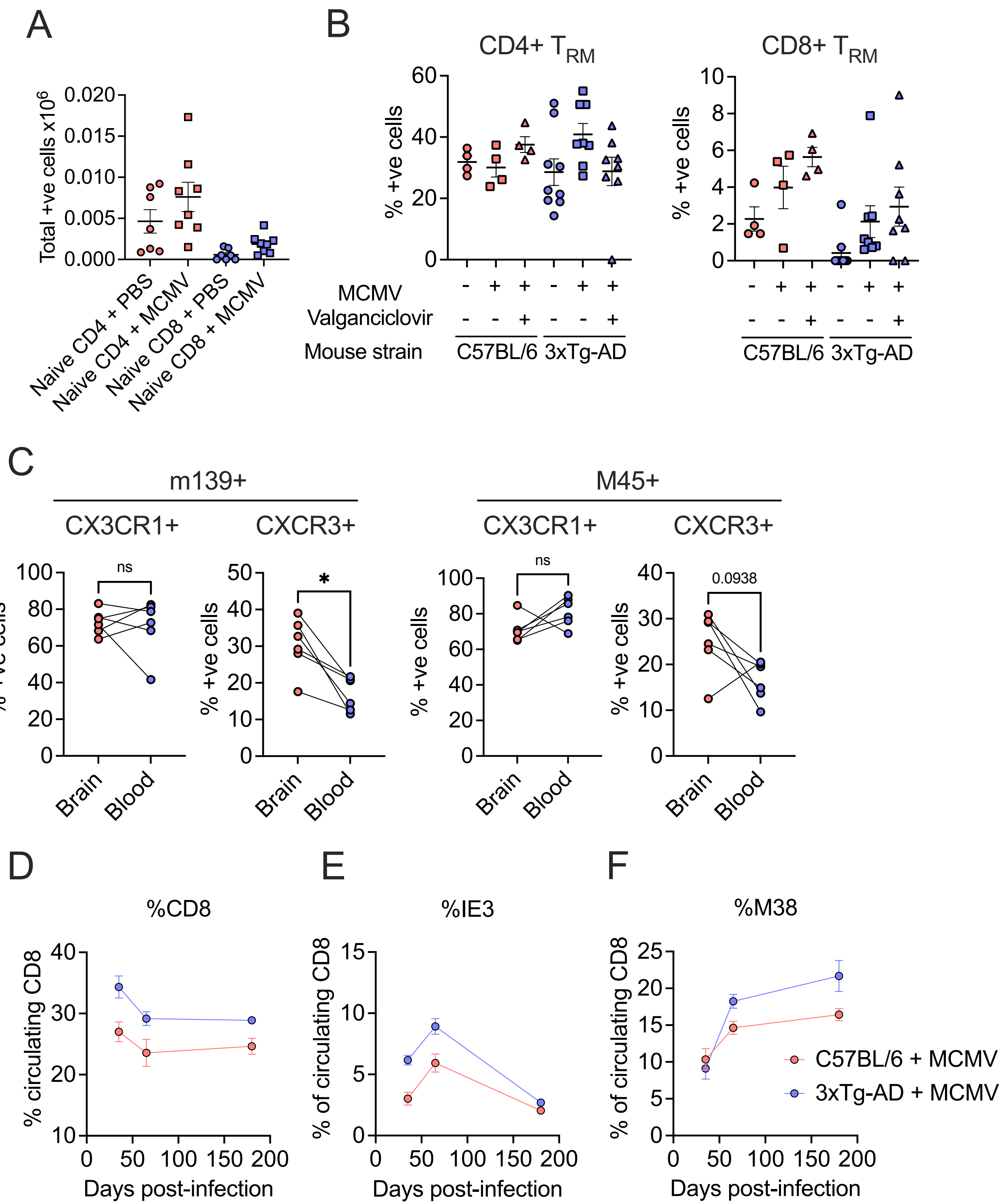

Supplemental Figure 5

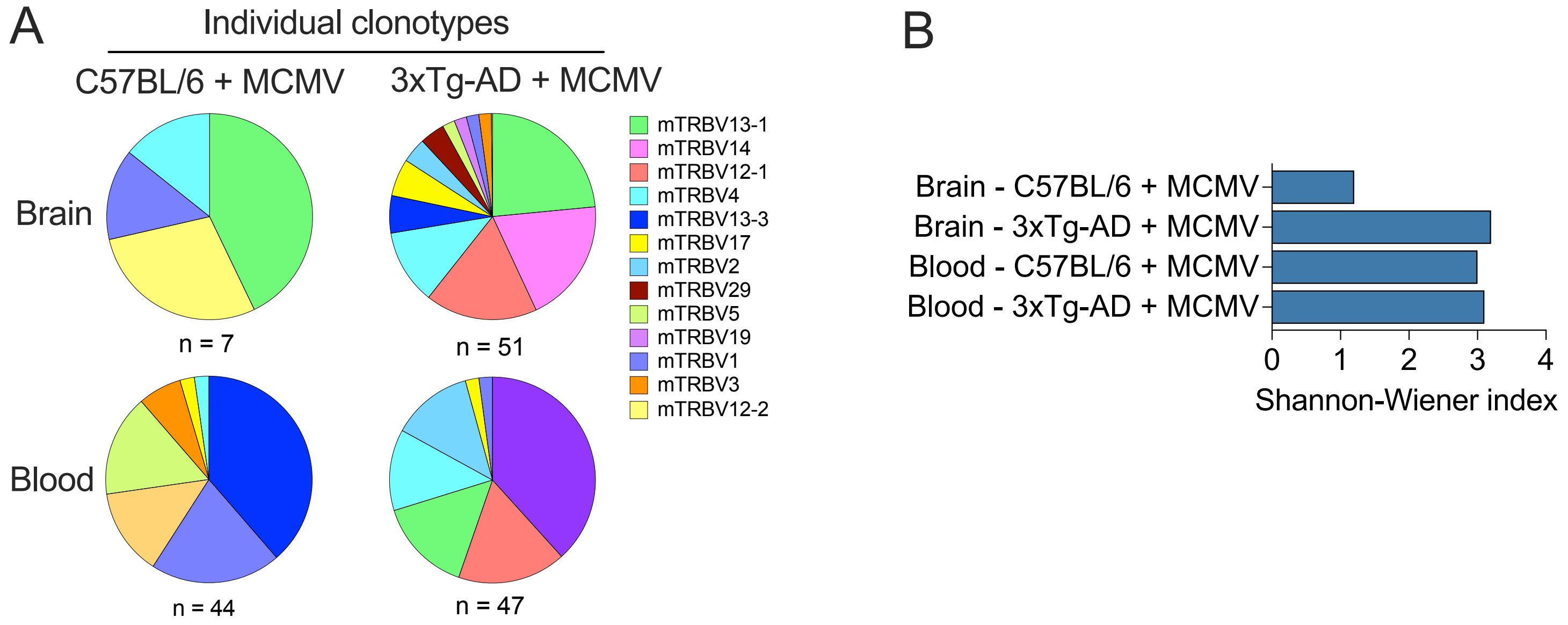

Supplemental Figure 6

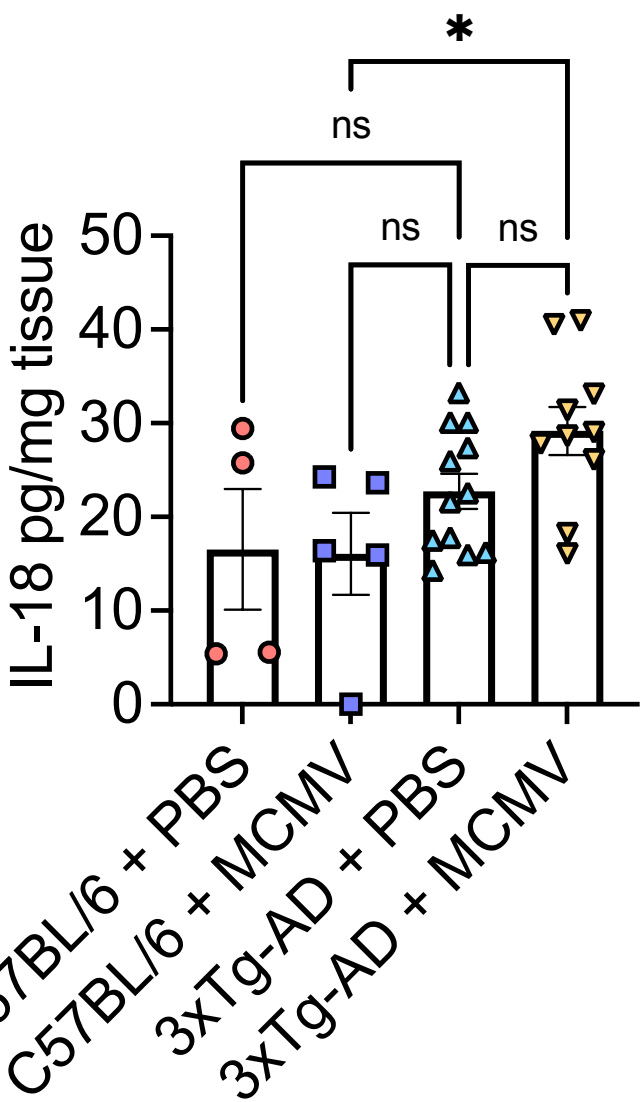

Supplement: Supplementary file 1 [file awag043_supplementary_data.pdf]
